# The processing of pseudoword form and meaning in production and comprehension: A computational modeling approach using linear discriminative learning

**DOI:** 10.3758/s13428-020-01356-w

**Published:** 2020-05-06

**Authors:** Yu-Ying Chuang, Marie Lenka Vollmer, Elnaz Shafaei-Bajestan, Susanne Gahl, Peter Hendrix, R. Harald Baayen

**Affiliations:** 1 Seminar für Sprachwissenschaft, Eberhard-Karls University of Tübingen, Tübingen, Germany; 2 Department of Linguistics, University of California at Berkeley, Berkeley, CA USA

**Keywords:** Auditory pseudowords, Auditory comprehension, Speech production, Linear discriminative learning, Morphology, Computational modeling

## Abstract

Pseudowords have long served as key tools in psycholinguistic investigations of the lexicon. A common assumption underlying the use of pseudowords is that they are devoid of meaning: Comparing words and pseudowords may then shed light on how meaningful linguistic elements are processed differently from meaningless sound strings. However, pseudowords may in fact carry meaning. On the basis of a computational model of lexical processing, linear discriminative learning (LDL Baayen et al., *Complexity, 2019*, 1–39, 2019), we compute numeric vectors representing the semantics of pseudowords. We demonstrate that quantitative measures gauging the semantic neighborhoods of pseudowords predict reaction times in the Massive Auditory Lexical Decision (MALD) database (Tucker et al., 2018). We also show that the model successfully predicts the acoustic durations of pseudowords. Importantly, model predictions hinge on the hypothesis that the mechanisms underlying speech production and comprehension interact. Thus, pseudowords emerge as an outstanding tool for gauging the resonance between production and comprehension. Many pseudowords in the MALD database contain inflectional suffixes. Unlike many contemporary models, LDL captures the semantic commonalities of forms sharing inflectional exponents without using the linguistic construct of morphemes. We discuss methodological and theoretical implications for models of lexical processing and morphological theory. The results of this study, complementing those on real words reported in Baayen et al., (*Complexity, 2019*, 1–39, 2019), thus provide further evidence for the usefulness of LDL both as a cognitive model of the mental lexicon, and as a tool for generating new quantitative measures that are predictive for human lexical processing.

**Electronic supplementary material:**

The online version of this article (10.3758/s13428-020-01356-w) contains supplementary material, which is available to authorized users.

## Introduction

Pseudowords such as , i.e. phonologically legal forms that are not in the lexicon of a given language,^1^ are used extensively in a wide variety of linguistic and psycholinguistic experiments. Typically, the purpose of including such items is to examine how the processing of meaningful words differs from that of strings of sounds or letters that are, by assumption, devoid of meaning. In research on speech perception, for example, pseudowords have been used to study phonological effects, such as phonological neighborhood density and phonotactic probability, on speech processing. Vitevitch and Luce (1998), using a shadowing task, found that while higher probabilities and denser neighborhoods were associated with longer naming response times for words, correlations became negative for pseudowords. Since pseudowords *ex hypothesi* lack semantics, the phonological effects observed on pseudowords are interpreted to occur at the sublexical level.

However, is the processing of pseudowords truly detached from the mental lexicon? What cognitive mechanisms underlie the comprehension and production of pseudowords? Current computational models of lexical processing provide limited insight into this question. In standard interactive activation models of visual (McClelland & Rumelhart, 1981) and auditory word recognition (McClelland & Elman, 1986), for example, there are no entries for pseudowords in the lexicon, reflecting the assumption that pseudowords do not appear in the lexicon and do not carry meaning. Bayesian word recognition models (Norris, 2006; Norris & McQueen, 2008) include mechanisms for modeling the behavior of pseudowords, in order to simulate the situation of encountering unknown words. Although in the latter model pseudowords find their way into the mental lexicon, very little can be said about their semantic make-up or their semantic relations with other words.

Some computational methods provide ways to study the semantics of pseudowords. For example, Marelli, Amenta, and Crepaldi (2014) and Amenta, Marelli, and Sulpizio (2017) investigate the degree of semantic similarity between a given word and other words that share orthographic or phonological subsequences. The meanings of pseudowords can also be estimated more directly. The triangle model (Harm & Seidenberg, 2004) dynamically computes the meaning of a word from its input codes. Using its networks as trained on words, it can in principle also estimate the meaning of a pseudoword, in the same manner as for a real word, although the amount of activation produced by pseudowords is reported to be substantially less than that produced by words (Harm & Seidenberg, 2004, p. 680–681).

More recently, Baayen, Chuang, Shafaei-Bajestan, and Blevins (2019) put forward the model of linear discriminative learning (LDL) for the mental lexicon. Just as in the triangle model, meaning is computed dynamically, rather than retrieved. However, the training algorithm behind LDL, detailed below, is much simpler than that of the triangle model. Baayen et al., (2019) show that LDL achieves high accuracy for both word comprehension and production. Furthermore, measures derived from LDL networks are highly predictive of behavioral data.

Cassani, Chuang, and Baayen (2019) is the first study that used LDL to investigate pseudowords. Taking the 16 pseudowords from the experiment of Fitneva, Christiansen, and Monaghan (2009) on children’s lexical categorization, Cassani et al., (2019) generated high-dimensional numeric representations for the semantics of pseudowords (henceforth semantic vectors) and calculated their correlation with the semantic vectors of real words as well as those of morphological functions. They showed that children’s responses could be accurately predicted in this manner.

In this study, we extend the line of pseudoword research to pseudoword auditory recognition, and from there to spoken production: If pseudoword meanings can be computed based on their forms, one can ask to what extent the production of pseudowords can be predicted from their (computed) meanings. Using the pseudoword data from the Massive Auditory Lexical Decision (MALD) database (Tucker et al., 2018), we conducted a large-scale study on auditorily presented pseudowords. As described in detail below, the MALD database comprises a set of recordings of spoken words and pseudowords, which we used as input for the LDL model to estimate semantic vectors for pseudowords. Moreover, as LDL can model not only comprehension but also production processes, we examined as well the model’s predictions concerning the pronunciation of pseudowords—specifically, their acoustic durations—on the basis of their semantic vectors. Below, we show that measures derived from both comprehension and production networks are all highly predictive of auditory lexical decision times (as a measure of comprehension), as well as of the spoken pseudoword durations (as a measure of speech production). In addition, when compared to the classical form-based measures such as phonological neighborhood density, the LDL measures together provide better prediction accuracy.

A substantial proportion of the pseudowords in the MALD database contains English inflectional suffixes, and hence are morphologically complex. LDL is constructed specifically for being able to process morphologically complex words, including out-of-vocabulary novel complex words. This in turn enables the model to capture in part the inflectional meanings of morphologically complex pseudowords. By way of example, a pseudoword ending in the exponent (e.g., ) is very likely to be interpreted as a certain action with the continuous aspect. In our model, the inflectional meaning of continuous emerges because the exponent will be mapped onto an area in semantic space where real words with the exponent are located.

The paper proceeds as follows. We begin by describing the architecture of the LDL model (Section “A blueprint of the mental lexicon using linear discriminative learning”) and the treatment of morphology in current computational models and in LDL (Section “Models of morphological processing”). We then present the methods (Section “Modeling auditory pseudowords”) and results (Section “Results”) of modeling the processing of auditory pseudowords with LDL. Finally, we discuss the results against the background of current models of speech production and comprehension, as well as their methodological and theoretical implications for research on lexical processing and morphological theory (Section “Discussion”).

## A blueprint of the mental lexicon using linear discriminative learning

**Fig. 1:**
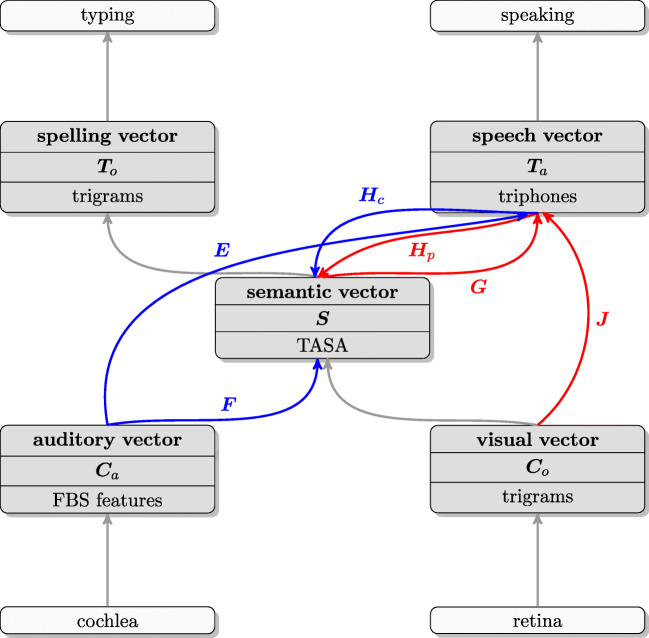
Overview of the discriminative lexicon. Input and output systems are presented in *light gray*, the vector representations characterizing the state of form and meaning subsystems are shown in *dark gray*. The vectors of individual words are brought together as the row vectors of the matrices ***C***_*o*_, ***C***_*a*_, ***S***, ***T***_*a*_, and ***T***_*o*_. *Arrows* represent linear mappings between vectors. Mappings relevant to the present study are labeled. Mappings in *red* (***J***, ***H***_*p*_, ***G***) represent networks involved in production, whereas mappings in *blue* (***F***, ***E***, ***H***_*c*_) represent networks involved in comprehension. The implementation is detailed in Section “Model definition and predictors derived from the model”

The computational model of linear discriminative learning, as laid out in Baayen et al., (2019), makes use of insights from machine learning, but uses implementations that are much simpler and linguistically transparent. The mental lexicon as modeled with LDL comprises five high-dimensional numeric vectors (shown in grey blocks in Fig. 1), each of which represents the state of a different subsystem.

1. The **visual vector** is a binary vector that specifies which letter trigrams are instantiated in the visual input. The length of an orthographic cue vector is equal to the number of different letter trigrams in the training data. Trigrams that are present are coded with 1, and those that are absent with 0. The visual vectors for the orthographic words in the training data are brought together as the row vectors of matrix ***C***_*o*_.^2^
2. The **auditory vector** is a binary vector specifying which acoustic features are present in a word’s audio signal. In both Baayen et al., (2019) and the present study, we used the frequency band summary (FBS) features developed by Arnold, Tomaschek, Lopez, Sering, and Baayen (2017), which will be described in more detail below (Section “From pseudowords’ audio files to pseudowords’ semantics”). Similar to the visual vector, the length of an auditory cue vector is equal to the number of different FBS features in the training data, and matrix ***C***_*a*_ has as its row vectors the auditory vectors of words’ speech tokens present in the training data.
3. The **semantic vector** represents a word’s meaning. Semantic vectors, known as embeddings in computational linguistics, can be derived in many different ways from corpora (see, e.g., Landauer & Dumais, 1997; Jones & Mewhort, 2007; Shaoul & Westbury, 2010; Mikolov et al., 2013).^3^ Following the method outlined in Baayen, Shaoul, Willits, and Ramscar (2016) and Baayen et al., (2019), we derived semantic vectors from the TASA corpus (Ivens and Koslin, 1991), the corpus that was used by Landauer and Dumais (1997) to construct the semantic vectors of latent semantic analysis. The semantic vectors of the words in the training data constitute the row vectors of matrix ***S***. Details about the method of calculating semantic vectors are provided in Section “Morphology with LDL”, where we explain how we constructed semantic vectors for morphologically complex words.
4. The **speech vector** is a binary vector indicating which triphones should be realized when a word is articulated. Again, the length of a speech vector is equal to the number of different triphones in the training data, presence is marked with 1 and absence with 0. The row vectors of matrix ***T***_*a*_ are the speech vectors of the words in the training data. In the LDL model, the triphone vectors serve two functions. On the one hand, for production, they represent abstract context-sensitive phonological targets that will further drive articulation.^4^ On the other hand, the triphone vectors also play a role in comprehension. (See the discussion of dual-route processing in visual and auditory comprehension in Section “From pseudowords’ audio files to pseudowords’ semantics”.)
5. The **spelling vector** specifies which letter triplets are present in a word that is to be written.

Note that this model does not make use of slot (or position) specific vectors. The visual, spelling, and speech vectors simply consist of indicator variables for the presence of letter or phone triplets. However, by using trigrams or triphones, time information is still implicitly coded in the order sequences (more details about finding the order of triphones are provided in Section “From pseudowords’ transcriptions to pseudowords’ forms”.) As will become clearer later, this implicit time information turns out to be sufficient for obtaining high-quality mappings to and from semantic vectors.

With respect to the mappings between vectors (represented by arrows in Fig. 1), here we implemented linear mappings. These are equivalent to networks with input and output units and no hidden layers (and no squashing functions). Effectively, this amounts to a multivariate multiple linear regression approach. These linear mappings can be learned incrementally using the update rule of Widrow and Hoff (1960). For computational convenience, in the present study we estimate mappings using the linear algebra of multivariate regression. Accordingly, each mapping is defined by a matrix ***A*** that transforms the row vectors in a matrix ***X*** into the row vectors of a matrix ***Y***; i.e., ***Y*** = ***XA***. As an example, consider the production network ***G*** (cf. Fig. 1): We can obtain this network by solving ***T***_***a***_ = ***SG***: $\boldsymbol {G} = \boldsymbol {S}^{-}\boldsymbol {T}_{a}$, where ***S***^−^ is the generalized inverse of ***S***. (We refer the interested reader to Baayen et al., (2019) for an informal introduction to the mathematical details.) Linear mappings are restricted in what they can accomplish, but with carefully chosen input and output representations, they can be surprisingly effective and even solve some non-linearly separable classification tasks (see Milin et al., 2017, for detailed discussion).

## Models of morphological processing

Roughly half of the words in MALD are inflected. Since the pseudowords are of similar build as the words, roughly half of the pseudowords are potentially inflected variants of a possible but non-existing English word. Therefore, following the lead of Harm and Seidenberg (2004), who discuss in detail how the triangle model performs for inflected words, in the present study we must ensure that our model handles morphologically complex words appropriately. Fortunately, the LDL model, which we use to study the processing of auditory pseudowords, is designed specifically to also produce and understand morphologically complex words. Since the way in which LDL achieves this differs substantially from the way in which standard approaches deal with morphology, in this section we first discuss the theoretical construct of the morpheme in linguistics. We then discuss interactive activation and connectionist computational models of morphological processing. Against this background, we then introduce how LDL handles complex words.

### The theoretical construct of the morpheme

Work on logic (Frege, 1879; Russell, 1905; 1942) has had a profound influence on formal linguistics, leading to the widespread belief that language is grounded in a homomorphism between a calculus (or symbolic algebra) of form units and a calculus based on semantic primitives (e.g., Montague, 1973; Hornstein, 1995). Accordingly, language is viewed as a compositional system, and research is aimed at finding the building blocks of form, the rules for putting these building blocks together, and their semantic correlates.

In linguistic morphology, the field of study that addresses the relation between words’ forms and their meanings, the idea that words can be decomposed into morphemes, defined as the smallest units of form that correspond to elementary semantic units, became influential in the middle of the previous century and dominated linguistics in the US in the 1940s and 50s. Blevins (2016) refers to this linguistic tradition, which sought to systematize the work of Leonard Bloomfield, as post-Bloomfieldian American structuralism. In this approach, the inflectional variants of the English verb *to walk*—*walks, walked, walking*—are taken to be composed of the stem *walk* and the morphemes *-s*, *-ed*, and *-ing*, which have as their semantic corollaries third person singular, past, and continuous tense. For languages with more complex morphology, such as Turkish, a form such as *evlerinizden*, ‘from your houses’, is analyzed as consisting of a noun stem *ev* (house), a plural suffix *-ler*, a possessive pronominal suffix *iniz* (your), and a postpositional suffix *den* (from). The perspective of generative linguistics on morphology, which builds on post-Bloomfieldian structuralism, is stated succinctly by Embick and Poeppel (2015): “language comprises a set of representations (e.g., ‘morpheme’) and computations (e.g., ‘concatenation’) whose formal properties are the object of research in (psycho)linguistic theory …” (p. 357).

The theoretical construct of the morpheme has been extremely influential in psychology and cognitive science, where it is widely believed that morphemes must exist in the mind (Butz & Kutter, 2016; Zwitserlood, 2018). In fact, the majority consensus in current research on morphological processing in the mental lexicon is that morphology is symbolic, localist, decompositional in comprehension, and compositional in production (see, e.g., Smolka et al., 2014; Rastle & Davis, 2008; Beyersmann, Casalis, Ziegler, & Grainger, 2015; Beyersmann et al., 2016; Dell, 1986; Levelt, Roelofs, & Meyear, 1999), a tradition that goes back to the seminal work of Forster (1976) and Taft and Forster (1975, 1976).

Yet, already in the 1950s, researchers in linguistics started to realize that many languages do not build their words in the simple way suggested by the above example from Turkish (Hockett, 1954). Although the words of just about any language can be analyzed into sequences of morphemes, as beads on a string, and fitted to the procrustean bed of compositionality, this seldom leads to deeper understanding. Many theoretical morphologists therefore regard the morpheme as an infelicitous technical construct (see, e.g., Matthews, 1974, 1991; Beard, 1977; Stump, 2001; Blevins, 2006). This line of work in linguistics has led to a series of experimental studies that challenge the primacy of decomposition (in comprehension) and concatenation (in production). These studies call attention to, for instance, the early effects of whole-word properties in both eye-tracking and lexical decision (see, e.g., Baayen, Dijkstra, & Schreuder, 1997; Feldman, O’Connor, & Moscoso del Prado, 2009; Kuperman et al., 2009; Schmidtke et al., 2017), and to phonetic properties of complex words that do not follow from the properties of their constituents (Kemps, Ernestus, Schreuder, & Baayen, 2005a, b; Pluymaekers, Ernestus, & Baayen, 2005; Kemps, Ernestus, Schreuder, & Baayen, 2004).

Several solutions have been put forward for addressing the many fundamental problems associated with the theoretical construct of the morpheme as minimal sign (see, e.g., Blevins, 2016; Chuang et al., 2019, for detailed discussion of the theoretical issues). One solution is to use a variety of formal mechanisms that whip a morphological system into the mold of an item-and-arrangement system such as exemplified by Turkish. An example is the analysis of Hebrew stem allomorphy proposed by McCarthy (1981), according to which the allomorphs *katab* (present) and *ktob* (past) are comprised of two morphemes, a root morpheme consisting of the consonants *ktb* and a vowel morpheme, *a-a* for present and *o* for past tense. For a critique, see Ussishkin (2005).

Another solution is to give up the idea that morphemes are linguistic signs and reconceptualize them as units of form only. Realizational theories of morphology, such as developed by Stump (2001), avoid the term ‘morpheme’ and use the term ‘exponent’ to refer to units of form expressing inflectional or derivational functions. Given a lexical meaning and a set of inflectional features (spelling out number, person, case, tense, etc.), rules and representations are set up that formalize how bundles of inflectional features are realized at the form level. The theory of distributed morphology (Halle and Marantz, 1993) takes inflectional features to be syntactic in nature, and ‘distributes’ these features to different parts of syntactic tree graphs. Unlike realizational theories, distributed morphology retains the term ‘morpheme’, but uses it to alternatively denote units of form and units of meaning, without positing one-to-one links between the two (Marantz, 2013).

Yet another approach in theoretical morphology, first proposed by Matthews (1974) and Matthews (1991) and subsequently developed further by Blevins (2003, 2016), is known as Word and Paradigm Morphology. Discarding morphemes and exponents altogether, this approach treats words as basic units for lexical processing. Instead of stringing up small pieces of form into words, the analogical relations within and across paradigms serve as basis for word formation.

### Computational models for morphological processing

In psychology, the two best known computational models for speech production adopted the realizational perspective on morphology. The form part of the morpheme-as-sign and its meaning part are assigned to different representational levels. The models of both Dell (1986) and Levelt et al., (1999) have nodes for concepts and inflectional features, and nodes for morphs. The latter are referred to as morphemes by Dell (1986) and as lexemes in WEAVER. Links between the semantic and form units of morphemes-as-signs have no special status in these models.

Two influential computational models for auditory word recognition, TRACE (McClelland and Elman, 1986) and Shortlist/Shortlist B (Norris, 1994; Norris & McQueen, 2008) do not address morphological processing. TRACE included only monomorphemic words, and the two Shortlist models treat morphologically complex words in exactly the same way as monomorphemic words, including both in a lexical list of target forms for recognition. Similar implementation is found in a more recently developed model, DIANA (Ten Bosch, Boves, & Ernestus, 2015). These models are therefore “full-listing” models, and cannot be expected to perform well for languages such as Turkish or Estonian, for which the probability of encountering out-of-vocabulary inflected forms is simply too high.

Turning to visual word recognition, the interactive activation model (IAM) (McClelland & Rumelhart, 1981) does not address morphologically complex words, and the same holds for the Bayesian reader model of Norris (2006). To our knowledge, the only study that extends the IAM to include the processing of morphologically complex words is the LEIA model proposed by Veríssimo (2018). This model adds a lemma level to the IAM, and partitions nodes at both the word form and lemma levels into two sets: stems on the one hand, and affixes on the other. Form nodes for stems have inhibitory connections between them, and so do the form nodes for affixes. There are no connections between stem forms and affix forms. Affix forms have excitatory connections to their corresponding lemmas (e.g., *ed* to past), and vice versa.

The architecture of the LEIA computational model, as well as the architecture of related (but unimplemented) models formulated at higher levels of abstraction such as the stem-based access model of Smolka, Zwitserlood, and Rösler (2007); Smolka, Preller, and Eulitz (2014), illustrate a problem that becomes substantially exacerbated once languages with more complex morphology than English are considered, namely, that a lot of engineering is required to make the system work properly. Unsurprisingly, morphological theories adopting morphemes or exponents as decompositional units have addressed exactly this question in considerable detail.

What morpheme-based theories, realizational theories, and distributed morphology have in common is a concern with setting up systems of rules and representations that relate sets of semantic and/or syntactic features to combinations of units of form. The bread and butter of morphological analysis then is to set up these formal systems in the simplest and most insightful way. Such systems typically require extensive use of exception features, and necessitate setting up inflectional classes for subsets of forms that pattern in the same idiosyncratic way. Linguistic morphology has made far more progress here than computational models in psychology. In linguistic morphology, detailed formal methods have been developed that cover a wide range of languages with diverse complex inflectional systems. Localist computational models in psychology, by contrast, have been almost exclusively concerned with English.

However, one area where psychology is far ahead of linguistics is in exploring how learning systems might capture morphological effects without requiring hand-crafting of rule systems and lexicons with exceptions and specialized features for inflectional classes that inform these rules. For comprehension, the triangle model (Seidenberg & McClelland, 1989) in the implementation of Harm and Seidenberg (2004) worked with localist semantic features for English noun plural, past tense, and third person singular inflections. The model learned to predict these features from distributed phonological representations, and presented with inflected pseudowords, the units for these inflectional features were selectively activated. The model successfully learned which semantic functions are realized in words’ forms, without having to define exponents for these functions.

Experimentally observed graded effects of form and meaning for derived words have also been explained within the general framework of the triangle model (Seidenberg & Gonnerman, 2000; Plaut & Gonnerman, 2000; Gonnerman, Seidenberg, & Andersen, 2007), although to our knowledge the model has never actually been used to simulate these effects. Likewise, morphological effects in Hebrew have been discussed from the perspective of distributed connectionist modeling (Velan, Frost, Deutsch, & Plaut, 2005).

For speech production, the recurrent network developed by Mirković, MacDonald, and Seidenberg (2005) for Serbo-Croatian noun paradigms implements a realizational model. Localist representations for lemma, number, case, and animacy were implemented in a network that was trained to produce the corresponding inflected phonological forms.

Although the parallel distributed processing (PDP) approach has been successful in calling attention to the importance of learning, the absence of further development and the absence in the literature of successful models for languages with complex inflectional systems suggest that about a decade ago the PDP approach had reached the limits of what it could technically accomplish.

In recent years, neural network technology has rapidly developed far beyond that available to the PDP programme. Artificial neural networks are now widely used in machine learning, and outperform almost all classical symbolic algorithms on tasks as diverse as playing Go (AlphaGo, Silver et al., 2016) speech recognition (deep speech, Hannun et al., 2014) and speech production (WaveNet, Oord et al., 2016). How far current natural language processing technology has moved away from concepts in classical (psycho)linguistics theory is exemplified by Hannun et al., (2014), announcing in their abstract that they “… do not need a phoneme dictionary, nor even the concept of a ‘phoneme’ ” (p. 1).

In the light of these advances in machine learning, several different research strategies suggest themselves. One is to adopt deep learning networks for predicting aspects of human lexical processing. Although prediction accuracy may be expected to be high, deep learning methods tend to be black boxes, in the sense that it is often impossible to understand how exactly they work. Another research strategy is to keep working with the classical linguistic approach to linguistic cognition, using rules and representations. The strategy followed within the LDL research programme strikes a middle ground, and strives to keep the mathematics of the model as transparent as possible while at the same time doing justice to the many insights offered by linguistic theories. However, LDL has in common with the PDP programme that it seeks to minimize the amount of hand-crafting for model building.

### Morphology with LDL

LDL is inspired by Word and Paradigm Morphology (Matthews, 1974; Blevins, 2016), and takes words to be the basic units for lexical processing. Knowledge of morphology is brought into the model through the semantic vectors. Below we provide further detail about how we derive semantic vectors from corpora. Here we first outline the way in which the semantic vectors for morphologically complex words are constructed. As a first step, we define a set of basic semantic units, henceforth lexomes. These lexomes fall into two subgroups, content lexomes on the one hand, and inflectional and derivational lexomes on the other hand. The content lexomes can be morphologically simple forms such as *hand*, but also complex words such as *government* or *handsome*. The inflectional lexomes represent inflectional functions such as number, tense, aspect, person, and voice, and the derivational lexomes function such as agent (*runner*), patient (*addressee*), and negation (*unkind*). Each lexome is paired with a semantic vector. Thus, a lexome can be understood as a pointer to a semantic vector (Milin et al., 2017), but also as a location in a high-dimensional semantic space.

The semantic vector of a monomorphemic word is identical to that of its corresponding lexome. The semantic vector of an inflected word is defined as the sum of the semantic vectors of its associated lexomes. For example, the semantic vector for the noun *walks*, $\overrightarrow {walks}$, is the sum of the semantic vectors of walk and plural, i.e., $\overrightarrow {walk} + \overrightarrow {plural}$. By contrast, for the verb *walks*, the semantic vector is given by $\overrightarrow {walk} + \overrightarrow {3sg} + \overrightarrow {present}$.

The semantic vectors that we used in the present study are those described in detail in Baayen et al., (2019). These vectors were constructed from the TASA corpus. The words in this corpus were first parsed into their lexomes. Inflected words were represented by their stem and sense-disambiguated labels for their inflectional functions. By using part of speech tagging (the TreeTagger of Schmid, 1995), we were able to determine whether a form such as *walks* was used as a verb or as a noun. If it was a verb, its lexomes were walk, 3sg and present, but if it was a noun, it was assigned the lexomes walk and plural. Irregular past tense forms such as *went* were given the lexomes go and past. Derived words, which involve word formation and hence typically have idiosyncratic meanings, were assigned a lexome for the (derived) stem and a lexome for the derivational function. Following Baayen et al., (2016) and Milin et al., (2017), we used naive discrimination learning (NDL) (Baayen, Milin, Filipović Durdević, Hendrix, & Marelli, 2011; Sering, Milin, & Baayen, 2018) to build semantic vectors. The Rescorla–Wagner update rule was applied incrementally to the sentences in the TASA corpus. For each sentence, the algorithm was given the task to predict the lexomes in that sentence from all lexomes present in that sentence. Thus, a given word in a sentence is also predicting itself. After going through all the sentences in the corpus, a 23,562 × 23,562 weight matrix ***A*** is obtained. This matrix specifies, for a given lexome at row *i*, the association strengths of this lexome with each of the other lexomes listed in the columns of the weight matrix. We set the main diagonal of the weight matrix to zero, as otherwise the row vectors of the weight matrix, which constitute our semantic vectors, would be dominated by the extent to which words predict themselves (see, Baayen et al., 2019, for discussion of the pros and cons—depending on the task—of semantic vectors obtained with or without setting the diagonal of the weight matrix to zero). Furthermore, given that the majority of weights in ***A*** are zeros, indicating no information contained, we further removed columns that have small variances (*σ* < 3.4 × 10^− 8^) in ***A***. The resulting matrix ***A*** is of dimension 23,562 × 5,030. For other ways of bringing in morphology into semantic vector space models, see Luong, Socher, and Manning (2013); Botha and Blunsom (2014); Qiu, Cui, Bian, Gao, and Liu (2014); Cotterell and Schütze (2015); Chen, Xu, Liu, Sun, and Luan (2015). The vectors that we used in the present study were constructed without word sense disambiguation. Improved vectors can be obtained when word sense disambiguation and named entity recognition is carried out in addition to a morphological analysis and part of speech tagging, as shown by Long (2018).

In order to ensure that the results reported below are not contingent on the specific way in which we calculated the semantic vectors, or on the TASA corpus, we also conducted the same analyses using word embeddings obtained with Word2Vec applied to a corpus of Tweets. As results are very similar, details are reported in the Appendix, and not further discussed below.

## Modeling auditory pseudowords

The following two sections present the methods and results of modeling comprehension and production of auditory pseudowords with LDL. Key questions of interest to us fall into three sets. First, how do we evaluate the semantics of pseudowords? For real words, to evaluate model performance, one compares a word’s predicted semantic vector with the semantic vector that served as that word’s gold standard during training. But for pseudowords, there is no gold standard semantic vector to be compared with. One possibility is to inspect the semantic neighborhoods of pseudowords. This helps us locate the position at which a pseudoword lands in the high-dimensional semantic space, as well as which words and how many words the pseudoword is semantically similar to. We then are able to use quantitative measures (e.g., semantic neighborhood density) to predict pseudowords’ acoustic durations and auditory lexicality response times, all of which are provided by MALD.

Second, since the LDL model comprises networks not only for comprehension but also for production, we can ask whether durations and reaction times depend also on how well the pseudoword form that the speaker produced, and that the listeners heard, matches with the form that is predicted by the pseudoword’s estimated semantic vector. Does it matter how strongly the top form candidate (which typically will not be an existing word) is supported by the pseudoword vector? Do the production and comprehension systems ‘resonate’, such that the correlation of the observed pseudoword semantic vector with the semantic vector predicted by top form candidate is predictive for reaction times and durations?

Third, as mentioned earlier, many pseudowords in the MALD database contain affixes. As morphological effects should emerge from the system even without having to parse pseudowords into pseudostems and real affixes, one would expect the semantic vectors of pseudowords that are generated by the model to be morphologically differentiated. Thus, the semantic vectors of pseudowords with affixes should be different from the semantic vectors of pseudowords without affixes. In addition, finer distinction in affixal meanings should be revealed as well. That is, among pseudowords with affixes, those with the same affixes should be semantically more similar than those with different affixes. Finally, semantic similarity is expected to be observed in relation to words as well. In this regard, the semantic vectors of pseudowords with affixes should also be closer to the semantic vectors of words with corresponding affixes than the semantic vectors of those without. Note that these predictions only apply to inflectional functions, but not to derivational ones, given that LDL makes a strict distinction between inflection and word formation. As a consequence, derived words have their own lexomes, which reflect their own semantic idiosyncrasies (e.g., a worker can denote a specific kind of bee). In this study, we therefore focus specifically on inflection, leaving the study of derived pseudowords to future research.

In the following sections, we first provide further details on the MALD database. We then discuss how the LDL mappings were set up, and introduce the predictors that we derived from the model. We then discuss the results obtained, focusing first on the semantics of inflected pseudowords, and then on the predictions for pseudoword duration and pseudoword reaction time.

### The MALD data

Pseudoword data was obtained from the MALD database (Tucker et al., 2018). This database provides auditory lexical decision responses to 26,793 words and 9592 pseudowords, collected from 231 monolingual English listeners, aged 17–29. All pseudowords are composed of phonotactically legal syllables. The majority of the pseudowords have fewer than four syllables (96%), and no pseudowords have more than seven syllables. The distribution of the number of syllables in pseudowords is similar to that of the MALD words. The pseudowords of the MALD database were created with the Wuggy generator (Keuleers and Brysbaert, 2010), such that one-third of subsyllabic constituents of the input words were exchanged for other phonotactically legal segments with similar transitional probabilities. Thus, if the words from which the pseudowords are derived come with affixes, there is a high probability that pseudowords also inherit affixal forms, for example, , and .

All words and pseudowords were recorded by one male native Canadian English speaker. Words were presented in their standard spelling, while pseudowords were presented in their IPA transcriptions. The speaker was experienced and trained in reading the IPA. The speaker pronounced each word once. Pseudowords, on the other hand, were produced with at least three repetitions, and the most fluent rendition was selected for the experiment.

We also made use of pseudoword measures provided by the MALD database. One of the predictors for the analyses of acoustic duration and reaction time was **phonological neighborhood density** (PhonND), defined as the number of words which have an edit distance of one phone (by addition, deletion, or substitution) from a pseudoword. PhonND has been reported to determine pseudoword processing to a substantial extent by previous studies (Vitevitch, Luce, Charles-Luce, & Kemmerer, 1997; Vitevitch & Luce, 1998, 1999).

Another crucial factor that has also been widely studied in the literature is phonotactic probability. Given that this information is not provided by the MALD database, we used the online phonotactic probability calculator developed by Vitevitch and Luce (2004) to obtain the mean **biphone phonotactic probability** (BiphProb) for each pseudoword. This measure also serves as one of the predictors for the analysis of duration and reaction time.

### Model definition and predictors derived from the model

This subsection first introduces the mappings (networks) required to obtain pseudoword form vectors from the visual vector of pseudowords’ IPA transcriptions. Subsequently, we introduce the mappings for proceeding from pseudowords’ auditory vectors to pseudowords’ semantics.

#### Initializing the model with real words

Before we can evaluate the model’s performance on pseudowords, we first have to train the model with the real words. We used the MALD words as the training data. Although MALD contains 26,793 words, we only used 19,412 words for training, as it is only for these words that semantic vectors were found, and hence could be constructed based on matrix ***A***. However, many of these words are morphologically ambiguous. For example, the word *walks* can either be the third-person-singular form of the verb *walk*, or the plural form of the noun *walk*. As the semantic vectors of the verb *walks* and the noun *walks* will be different, due to different inflectional vectors being added to the semantic vector of the base word (cf. Section “Morphology with LDL”), the actual number of semantic vectors that we considered was not 19,412, but 23,637. Since in tasks such as single auditory word recognition, no contextual information is available for sense disambiguation, we took all possible meanings of an inflected word into account. For each of the mappings in Fig. 1, we obtained the mappings by solving the pertinent equations using the vectors for real words as row vectors of the relevant matrices. In the following subsections, we provide further detail about these mappings and how they were used for generating pseudoword vectors.

#### From pseudowords’ transcriptions to pseudowords’ forms

For the speaker in the experiment, the task is similar to a pseudoword naming task. An important difference with standard word naming is that the speaker had to pronounce the pseudowords at least three times. Ignoring this difference, three networks are involved during pseudoword production. In Fig. 1, these networks are highlighted in red.

The first network ***J*** maps a visual vector with the IPA trigrams (a row vector of ***C***_*o*_) to the corresponding speech vector of triphones (a row vector of ***T***_*a*_). The 23,637 × 8,601 matrix ***C***_*o*_ with words’ IPA form vectors specifies with 0/1 coding for each of the 23,637 inflectionally distinct (real) words in the MALD which of the 8601 possible IPA letter triplets it contains. The row vectors of the 23,637 × 8601 matrix ***T***_*a*_ specify which triphones are present in a word. For *walking* , for example, the triphones are and , with # indicating word boundaries. Given the one-to-one mapping between IPA trigrams and triphones, the mapping ***J***, obtained by solving ***C***_*o*_***J*** = ***T***_*a*_, is almost completely error-free. Given ***J***, the estimated speech vectors of pseudowords, the row vectors of $\hat {\boldsymbol {T}}_{a}^{(\text {pw})}$, are obtained by multiplication with the 9,592 × 8,601 IPA trigram matrix for the pseudowords $\boldsymbol {C}_{o}^{(\text {pw})}$:

1 $$ \boldsymbol{C}_{o}^{(\text{pw})} \boldsymbol{J} = \hat{\boldsymbol{T}}_{a}^{(\text{pw})}. $$

Although $\hat {\boldsymbol {T}}_{a}^{(\text {pw})}$ is a real-valued matrix with the predicted degree of support for each pseudoword’s triphones, the correlations of the row vectors of $\hat {\boldsymbol {T}}_{a}^{(\text {pw})}$ with the binary, true speech vectors of the pseudowords was 0.98 on average. Given that the triphones that truly exist in each pseudoword are also the most activated ones in $\hat {\boldsymbol {T}}_{a}^{(\text {pw})}$, we therefore used the binary triphone speech vector $\boldsymbol {T}_{a}^{(\text {pw})}$, instead of the estimated one ($\hat {\boldsymbol {T}}_{a}^{(\text {pw})}$) as input for subsequent networks. This procedure is also justified by unclarity as to how exactly to model the selection of the best-sounding pseudoword realization reported in the MALD from the three or more pronunciations that the speaker realized.

The network ***H***_*p*_ takes a speech vector of triphones and maps it onto a semantic vector. The semantic vectors of the words, constructed from the lexome matrix ***A*** as described in Section “Morphology with LDL”, were brought together as the row vectors of a matrix ***S*** of dimension 23,637 × 5030. The mapping ***H***_*p*_ is calculated by solving ***T***_*a*_***H***_*p*_ = ***S*** for words. The semantic vectors for the pseudowords (the row vectors of the semantic matrix $\hat {\boldsymbol {S}}_{0}$) follow as the product of $\boldsymbol {T}_{a}^{(\text {pw})}$ and ***H***_*p*_:

2 $$ \boldsymbol{T}_{a}^{(\text{pw})} \boldsymbol{H}_{p} = \hat{\boldsymbol{S}}_{0}.  $$

When speech production is driven not by visual input, but by internal conceptualization, a semantic vector ***s*** (a row vector of ***S***) is mapped by the network ***G*** onto a speech vector. The matrix ***G*** is calculated by solving ***SG*** = ***T***_*a*_ for words. A vector ***t***_*a*_ in ***T***_*a*_ represents the amount of support that triphones receive from the corresponding semantic vector ***s*** in ***S***.^5^ For pseudowords, we consider the possibility that a predicted semantic vector $\hat {\boldsymbol {s}}_{0}$ is mapped by the network ***G*** back onto a speech vector $\hat {\boldsymbol {t}}_{0}$:

3 $$ \hat{\boldsymbol{s}}_{0} \boldsymbol{G} = \hat{\boldsymbol{t}}_{0}. $$

The predicted pronunciation $\hat {\boldsymbol {t}}_{0}$ is of interest as the extent to which it deviates from the actual pronunciation may affect acoustic durations and reaction times. However, by itself, $\hat {\boldsymbol {t}}_{0}$ is just a vector of reals that define the degree of support coming from the semantics for each of the triphones known to the model. Typically, only a small minority of triphones receives strong support. In other words, $\hat {\boldsymbol {t}}_{0}$ is not an unambiguous representation of a word’s form. Fortunately, triphones contain implicit order information—*abc* and *bcd* can be joined into *abcd* but *pqr* and *qzx* cannot be merged—and hence can be strung together into sequences of phones, i.e., candidates for production, albeit with varying support from the semantics. The speak function from the WpmWithLdl R package (Baayen, Chuang, & Heitmeier, 2018b) derives such strings by first placing triphones as vertices in a directed graph, with directed edges connecting mergeable triphones (e.g., *abc* and *bcd*). Words can now be conceptualized as paths in this triphone graph. Algorithms from network science, taken from the igraph package for R (Csardi & Nepusz, 2006), are used to enumerate possible paths. To keep computational costs down, before calculating possible paths, the graph is thinned by removing edges with little support from the semantics. The threshold value that we use for removing edges was 0.1, its default value as used in several other studies (Baayen et al., 2019; Chuang et al., 2019; Baayen et al., 2018a). The speak algorithm then searches for all possible paths in the graph that start with an initial triphone (#xx) and end with a final triphone (xx#).

**Fig. 2:**
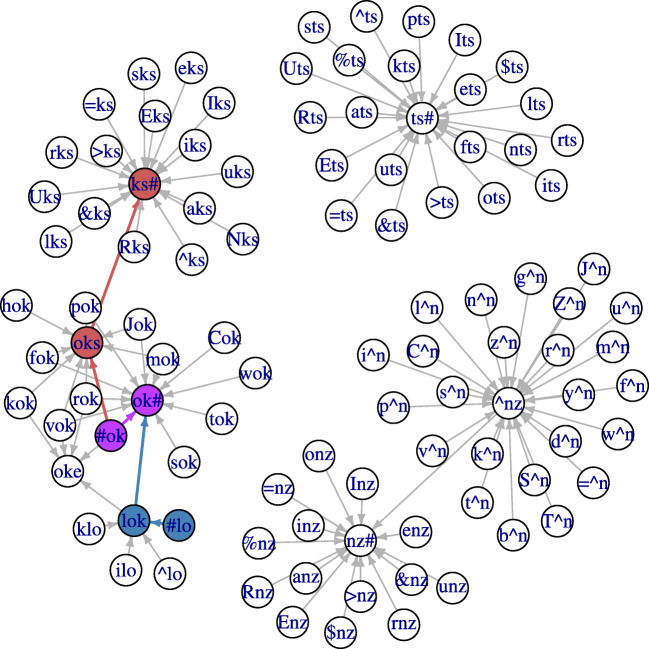
The (thinned) triphone graph highlighting three candidate forms [lok] (*blue*), [oks] (*red*), and [ok] (*purple*) for the target pseudoword [loks]

By way of example, for the pseudoword [loks], after removing the triphones (vertices) with little semantic support, the trimmed set contains 101 triphones. Two of them are initial triphones (#lo, #ok), and four of them are final triphones (ks#, ok#, nz#, ts#). The remaining 95 triphones are word-internal triphones (e.g., iks, aks, rok, oke, inz, uts,...). The 101 vertices and legitimate connections between them (edges) are represented as circles and arrows respectively in Fig. 2. Although there are often multiple edges between vertices, only three paths are found, as a legitimate pronounceable form needs to begin with an initial triphone (e.g., #lo) and end with a final triphone (e.g., ks#). These three paths are #lo→lok→ok# (marked in blue), #ok→oks→ks# (marked in red), and #ok→ok# (marked in purple). For the targeted form [loks] to be detected, the path #lo→lok→oks→ok# has to be available. However, the critical edge lok→oks in this path is not in the graph: it is a novel transition that is absent in the training data of real words. This leaves us with three candidate forms for this pseudoword, which are [lok], [oks], [ok]. For details of this path-searching algorithm for speech production, see Baayen et al., (2018a).

Let $\mathcal {P}$ denote the set of triphone paths returned by the speak function for an estimated semantic vector $\hat {\boldsymbol {s}}_{0}$. For each path $p \in \mathcal {P}$ there is a corresponding 0/1 triphone vector ***p***. Each of these triphone vectors is multiplied with ***H***_*p*_ to obtain a corresponding estimated semantic vector $\hat {\boldsymbol {s}}_{0}^{\prime }$:

4 $$ \hat{\boldsymbol{s}}_{0}^{\prime} = \boldsymbol{p} \boldsymbol{H}_{p}. $$

We refer to the set of vectors $\hat {\boldsymbol {s}}_{0}^{\prime }$ as $\mathcal {S}$. From this, we derived two measures that, as we shall see below, are predictive for both acoustic durations and auditory lexical decision latencies.

- **Average Levenshtein Distance of Candidates** (ALDC): The average Levenshtein distance of all candidate productions from the true pronunciation of a given pseudoword as provided in the MALD. Denoting the form (path) of the pseudoword as produced by the speaker by *π*, we have that
  5 $$ \text{ALDC} = {{\sum}_{i} \text{Levenshtein}(p_{i} \in \mathcal{P}, \pi) \over |\mathcal{P}|}. $$
  For the present example, the candidate forms $\mathcal {P}$ for the pseudoword *loks* are *lok*, *oks*, and *ok*, and the Levenshtein distances of each candidate from the pseudoword gold standard pronunciation are 1, 1, 2, respectively. The ALDC is thus 1.33. Note that in cases where no candidate forms are generated by the model, this measure is equal to the number of phones of the pseudoword. Candidate forms such as *lok*, *oks*, and *ok* are conceptually similar to the phonological neighbors of standard production models, and the ALDC measure is therefore conceptually similar to classical measures of phonological neighborhood density. Larger ALDC values indicate that the candidate forms are very different from the intended pronunciation, indicating a sparse form neighborhood. In the general discussion, we return to the question of how these neighborhood effects can be understood within our theoretical framework, as in this framework exemplars do not exist independently—all they do is leave traces in the mappings.
- **Semantic Correlation of Predicted Production** (SCPP): The maximum of the correlations between the semantic vector $\hat {\boldsymbol {s}}_{0}$ predicted from the speech vector and any of the semantic vectors $\hat {\boldsymbol {s}}_{0}^{\prime } \in \mathcal {S}$ generated from the candidate forms:
  6 $$ \text{SCPP} = \underset{i}{\text{argmax}}\ r(\hat{\boldsymbol{s}}_{0}, \hat{\boldsymbol{s}}_{0_{i}}^{\prime} \in \mathcal{S}). $$
  For the pseudoword [loks], the correlations of its semantic vector with the candidate forms are 0.64 for [lok], 0.47 for [oks], and 0.01 for [ok]. The SCPP is hence the correlation between the semantic vectors of [loks] and [lok]. When no predicted forms are generated by the model, this measure will be 0. The SCPP is higher when the semantics of the generated form better approximate the generated meaning.^6^

#### From pseudowords’ audio files to pseudowords’ semantics

Thus far, we have focused on the speaker. We now consider the modeling of how listeners understand pseudowords. For modeling auditory comprehension, we need form vectors that specify key properties of the audio signal. These form vectors are brought together in the matrices ***C***_*a*_ for words and $\boldsymbol {C}_{a}^{(\text {pw})}$ for pseudowords. As acoustic features we made use of the frequency band summary (FBS) features developed by Arnold et al., (2017). FBS features are summaries of the spectral information embedded in the speech signal. A word is first divided into chunks at the positions of the minima of the Hilbert-transformed envelope of the waveform. Within each chunk, consecutive power spectra of 5-ms windows are taken and then mapped onto 21 frequency bands on the MEL-frequency scale. Intensities in these frequency bands are subsequently discretized into five levels, and the distribution of the intensities in a band are then summarized in a FBS feature which brings together the initial and final intensity, maximum and minimum intensity, and median intensity. By way of example, the feature band1-start1-median2-min1-max4-end2-part1 specifies that for the first frequency band (band1) of the first chunk of the word (part1), the intensity of the initial window is 1 (start1), that of the final window is 2 (end2), and that the median, minimum, and maximum intensities are 2, 1, and 4 (median2, min1, max4) respectively. We extracted the FBS features from the audio files of the MALD database with the AcousticNDLCodeR package (Arnold, 2017). The total number of different FBS features extracted for the MALD words was 26,336. The 23,637 × 26,336 auditory matrix ***C***_*a*_ defines for each word (rows) which FBS features (columns) are present. Since each form vector in ***C***_*a*_ has a corresponding semantic vector in ***S***, we can again use a straightforward linear mapping to project acoustic vectors into semantic space (the ***F*** mapping in Fig. 1). The mapping ***F*** is obtained by solving ***C***_*a*_***F*** = ***S*** for words. Let the 9,592 × 26,336 matrix $\boldsymbol {C}_{a}^{(\text {pw})}$ contain as its row vectors the indicators for the FBS features of the 9,592 pseudowords.^7^ The semantic vectors estimated for pseudowords are summarized by a 9,592 × 5,030 matrix $\hat {\boldsymbol {S}}_{1}$:

7 $$ \boldsymbol{C}_{a}^{(\text{pw})} \boldsymbol{F} = \hat{\boldsymbol{S}}_{1}.  $$

For visual word recognition, Baayen et al., (2019) found that a dual-route setup, with a direct route straight from orthographic vectors to semantic vectors, and an indirect route going from orthographic vectors to triphone vectors and from the triphone vectors to the semantic vectors, afforded greater precision (see Wong and Chen, 1999; Perrone-Bertolotti et al., 2012; Newman et al., 2012; Jared, Ashby, Agauas, & Levy, 2016; Bitan, Kaftory, Meiri-Leib, Eviatar, & Peleg, 2017; Jared & O’Donnell, 2017; Amenta et al., 2017, for detailed discussion of dual routes in reading). This result led us to inquire whether a second, indirect route, would also enhance model performance for auditory comprehension. We designed this second route as follows: first, the acoustic cue vector in ***C***_*a*_ is mapped onto its corresponding triphone vector in ***T***_*a*_. Subsequently this vector in ***T***_*a*_ is mapped onto a semantic vector in ***S***. The two mappings required for this indirect route are given by the matrices ***E*** and ***H***_*c*_ in Fig. 1. The mappings ***E*** and ***H***_*c*_ are obtained by solving ***C***_*a*_***E*** = ***T***_*a*_ and ***T***_*a*_***H***_*c*_ = ***S***. Given these mappings for words, the semantic vectors $\hat {\boldsymbol {S}}_{2}$ predicted for pseudowords by the indirect route are calculated as follows:

8 $$ \boldsymbol{C}_{a}^{(\text{pw})} \boldsymbol{E} \boldsymbol{H}_{c} = \hat{\boldsymbol{S}}_{2}.  $$

In what follows, we use the notation $\hat {\boldsymbol {s}}_{1}$ for a row vector of $\hat {\boldsymbol {S}}_{1}$ (direct route) and $\hat {\boldsymbol {s}}_{2}$ for a row vector of $\hat {\boldsymbol {S}}_{2}$ (indirect route).

**Fig. 3:**
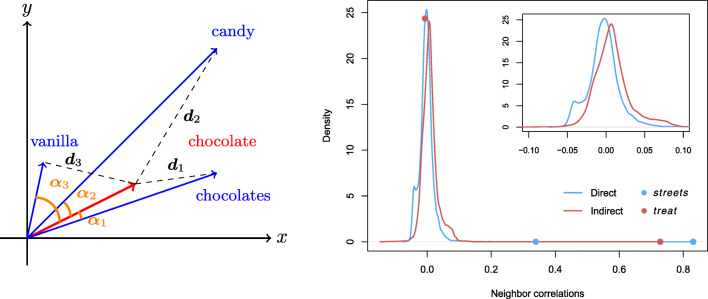
*Left panel*: graphical illustration of angles and distances for the semantic neighbors of *chocolate*; *right panel*: estimated probability density functions for the correlations of *street* with other words for the direct route (*in blue*) and the indirect route (*in red*). The smaller inset plot shows the same densities restricted to correlations ranging between – 0.1 and 0.1, to highlight the difference between the two densities. The dots on the density curves indicate the correlations of *street*’s two semantic vectors with the semantic vectors of *streets* and *treat*, the closest neighbors for the direct and the indirect routes. The *blue dot* at correlation 0.34 denotes the correlation of *street* and *streets* according to the indirect route

From this comprehension model, we derived further measures to quantify semantic relations between pseudowords and real words. To gauge semantic similarity, traditionally it is common to work with the angle between the semantic vectors of words, using either the cosine similarity or correlation measure. In addition to angle measures, the relation between two semantic vectors can be gauged by their proximity as well. For the semantic vectors that we used, which are not normalized for length, a measure of proximity, such as the Euclidian distance, is potentially informative. To illustrate this, the left panel of Fig. 3 presents three semantic neighbors of the word *chocolate*, whose semantic vectors are either highly correlated with the semantic vector of *chocolate* (i.e., small angles) or in its vicinity (i.e., short distance). These three word neighbors are *chocolates*, *candy*, and *vanilla*. The angles between each of their semantic vectors and the semantic vector of *chocolate* are denoted by *α*_1_, *α*_2_, *α*_3_, and the Euclidean distances from each of them to *chocolate* are denoted by *d*_1_, *d*_2_, and *d*_3_ respectively. As can be seen, the plural form *chocolates* is semantically closest to *chocolate*, given that both *α*_1_ and *d*_1_ are the smallest among the three. Interestingly, the remaining two words, *candy* and *vanilla*, are closely related to *chocolate* in different ways. While *candy* has a smaller angle with *chocolate* than *vanilla* does (*α*_2_ < *α*_3_), *vanilla* is however closer to *chocolate* in distance than *candy* (*d*_2_ > *d*_3_). In fact, it seems that the angle and distance measures have brought together different groups of semantic neighbors. For *chocolate*, according to the angle measure, its most correlated words are *chocolates*, *candy*, *cookie*, *butter*, *cream*, and *cake*. According to the distance measure, words that are nearest to *chocolate* are *chocolates*, *vanilla*, *frosting*, *peppermint*, *lemonade*, and *muffin*. Except that the plural form *chocolates* is listed as top in both measures, it appears that the two measures are capturing different semantic relations. Exactly what semantic relations are gauged by angle and distance measures as applied to the present semantic vectors is beyond the scope of this study. In what follows, we will see that both angle-based and distance-based measures are informative about the lexical processing of the pseudowords.

An issue deserving further scrutiny is how the semantic predictions generated by the direct and the indirect routes differ. For the MALD words, the correlations of the semantic vectors generated by the two routes are generally high ($\bar {r} = 0.73$). Upon closer inspection, when the two-route correlation is low, it usually can be traced to the phonological aspect of the indirect route having been foregrounded. The right panel of Fig. 3 plots the distributions of the correlations between the word *street* and all the other words in the training data, by means of the corresponding estimated probability density functions. The blue line indicates the correlations calculated between the semantic vector generated by the direct route and the (gold standard) semantic vectors of all the other words, whereas the red line indicates the correlations calculated with the semantic vector generated by the indirect route. The correlation between the semantic vectors derived from the two routes is 0.39, but the density curve for the direct route is shifted to the left compared to the curve of the indirect route (*p* < .00001, two-sample Kolmogorov–Smirnov test).

For the direct route, the most correlated semantic neighbor is *streets*, followed by *alley*, *lane*, *road*, and *avenue*. As to the indirect route, among the most correlated words we find *treat*, *treats*, *treated*, *streets* and *greet*. Since the second half of the indirect route maps high-level phonological representations (***T***_*a*_) to semantics ***S***, in this case the predicted semantics is influenced more by words’ canonical phonological forms. Given that the two routes predicted exactly the same semantic vectors for 4.5% of the MALD words (*r* = 1), and given that half of the words have a two-route correlation over 0.8, it is clear that the effect of canonical phonological forms is not across-the-board. Nevertheless, because the indirect route is forced through a layer of more abstract phonological triphones, the emergence of semantic neighbors that are more similar in their phonological form is to be expected.

In total, four measures were derived from the comprehension networks. These include a distance measure (EDNN), an angle measure (NNC), a density measure (ALC), and a two-route measure (DRC). Except for the last measure, we calculated all measures for both the semantic vectors derived from the direct route ($\hat {\boldsymbol {s}}_{1}$) and for those derived from the indirect route ($\hat {\boldsymbol {s}}_{2}$). In what follows, we present the formal definitions only for the direct route. Those for the indirect route can be obtained by substituting $\hat {\boldsymbol {s}}_{2}$ for $\hat {\boldsymbol {s}}_{1}$.

- **Euclidean Distance from Nearest Neighbor** (EDNN): The Euclidean distance from the semantic vector $\hat {\boldsymbol {s}}_{1}$ produced by the direct route to its closest semantic word neighbor:
  9 $$ \text{EDNN} = \underset{i}{\text{argmin}}\ \text{dist}(\hat{\boldsymbol{s}}_{1}, \boldsymbol{s}_{i}). $$
  Distances were calculated using the FNN package of R (Beygelzimer et al., 2018).
- **Nearest Neighbor Correlation** (NNC): The maximum of the correlations between a pseudoword’s estimated semantic vector and words’ semantic vectors:
  10 $$ \text{NNC} = \underset{i}{\text{argmax}}\ \text{r}(\hat{\boldsymbol{s}}_{1}, \boldsymbol{s}_{i}). $$
  We used the Pearson correlation rather than the cosine similarity. As the two are strongly correlated (*r* = 0.99), and as the correlation measure is the cosine of the angle of centered vectors, the two measures can be interchanged without affecting the results. The NNC is high when the angle between the two vectors is small and the pseudoword’s meaning is similar to that of a real word.
- **Average Lexical Correlation** (ALC): The mean of the correlations of a pseudoword’s estimated semantic vector with each of the words’ semantic vectors. Denoting the number of different word tokens by *v*, we have:
  11 $$ \text{ALC} = {{\sum}_{i=1}^{v} \text{r}(\hat{\boldsymbol{s}}_{1}, \boldsymbol{s}_{i}) \over v}. $$
  Higher values of ALC indicate that a pseudoword vector has “landed” in a denser semantic neighborhood.
- **Dual Route Consistency** (DRC): The correlation between the semantic vector estimated from the direct route and that from the indirect route :
  12 $$ \text{DRC} = \text{r}(\hat{\boldsymbol{s}}_{1}, \hat{\boldsymbol{s}}_{2}). $$
  When the DRC is higher, the semantic vectors produced by the two routes are more similar to each other.

**Fig. 4:**
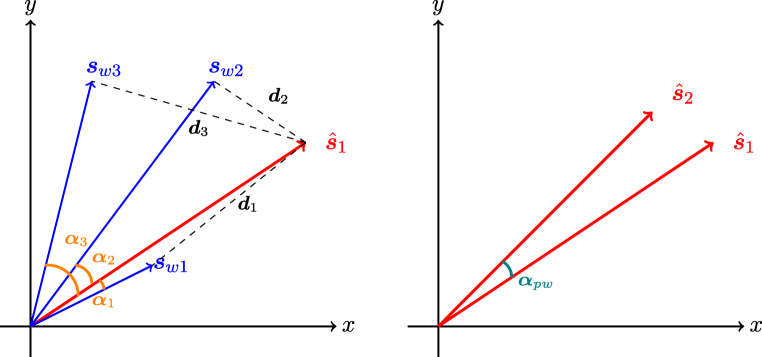
A pseudoword’s estimated semantic vector derived from the direct route ($\hat {\boldsymbol {s}}_{1}$) and the semantic vectors of three word neighbors (***s***_*w*1_, ***s***_*w*2_, ***s***_*w*3_) are shown in the *left panel*. The distances between the pseudoword from the three words are ***d***_1_, ***d***_2_, and ***d***_3_ respectively, and the angles are indicated by *α*_1_, *α*_2_, and *α*_3_. The *right panel* shows the estimated semantic vectors derived from the direct route ($\hat {\boldsymbol {s}}_{1}$) and the indirect route ($\hat {\boldsymbol {s}}_{2}$), and the angle *α*_*pw*_ between them

Figure 4 provides an illustration of how these measures are calculated. The left panel shows the semantic vectors of a pseudoword ($\hat {\boldsymbol {s}}_{1}$) and three real words (***s***_*w*1_, ***s***_*w*2_, ***s***_*w*3_). Given that the pseudoword is the closest to ***s***_*w*2_ in distance, for this pseudoword EDNN = ***d***_2_. With respect to the angles, let *f*(*α*) denote the cosine of the angle or the correlation between the two vectors. Then ALC = (*f*(*α*_1_) + *f*(*α*_2_) + *f*(*α*_3_))/3, and as $\hat {\boldsymbol {s}}_{1}$ and ***s***_*w*1_ have the smallest angle, NNC = *f*(*α*_1_). Finally, for DRC (right panel), as it is the measure of the angle between the semantic vectors estimated via the two routes, DRC = *f*(*α*_*pw*_) in this case.

It is worth noting that morphological information is embedded in the semantic vectors of words (cf. Section “Morphology with LDL”). When the semantic vectors for pseudowords are generated (via Eqs. 7 and 8 for the direct and indirect route respectively), the predicted morphological information for pseudowords will be in their semantic vectors as well, without the necessity of doing any morphological parsing. The reason for this is that the mappings from form to meaning as established for real words are sensitive to morphology, albeit indirectly. Through the same mappings, an auditory pseudoword ending in , for instance, will be mapped onto a general area of semantic space where real words with the exponent are located. Thus, when the measures described above are calculated, morphological information of words and pseudowords will be both taken into account.

## Results

For the analyses, we included only pseudowords with correct responses in MALD (i.e., those pseudowords that were rejected as words). Nineteen pseudowords were excluded because they received unanimous “word” responses. This left us with 9573 pseudowords. In what follows, we first examine the effect of inflectional affixes in pseudowords on their semantics. This analysis clarifies the consequences of morphological complexity according to our model. The next two sections report the structure on pseudoword duration and response time. We are interested in the extent to which the measures derived from the model can account for pseudoword production and perception, and how their predictability is when compared to some classical form-based measures such as neighborhood density and biphone phonotactic probability.

### Pseudoword morphology

As mentioned above, the pseudowords in MALD are created based on real words. If the base real word is an inflected word, then the corresponding pseudoword is very likely to carry the inflectional exponent as well. However, since the base real words are not available to us, we therefore labeled the inflectional functions of the pseudowords according to their forms by ourselves, using a combination of scripting and hand-correction. For example, the pseudoword looks like a verb with the inflection of continuous aspect, and is reminiscent of an adjective in its superlative form^8^. In total, seven inflectional functions were identified (following Baayen et al., 2019): comparative, superlative, continuous, past, perfective, person3, and plural. However, due to form similarity and the lack of contextual information, past and perfective cannot be distinguished from each other. The same happens for person3 and plural, as there is no way to tell which inflectional function a pseudoword has solely based on its inflected form. We therefore combined the ambiguous inflectional functions and partitioned the inflected pseudowords into five sets, the distribution of which is shown in Table 1. It turns out that nearly half of the pseudowords end in what could be an inflectional exponent of English. Note that the 110 pseudowords with the superlative form could possibly also be analyzed as realizing past/perfective (e.g., as an inflected variant of the verb ). These 110 pseudowords are thus included in both the superlative and the past/perfective sets.

**Table 1:** The distribution of inflected pseudowords in MALD

	# of pseudowords
comparative	481
superlative	110
continuous	722
past/perfective	1015
person3/plural	1773
Uninflected	5581

Even though the inflectional functions were not specified explicitly by means of form units when the semantic vectors of pseudowords were estimated, we expected that pseudowords labeled with the same inflectional function should be semantically similar to each other, and meanwhile semantically distinct from those labeled with different functions or uninflected pseudowords.

**Fig. 5:**
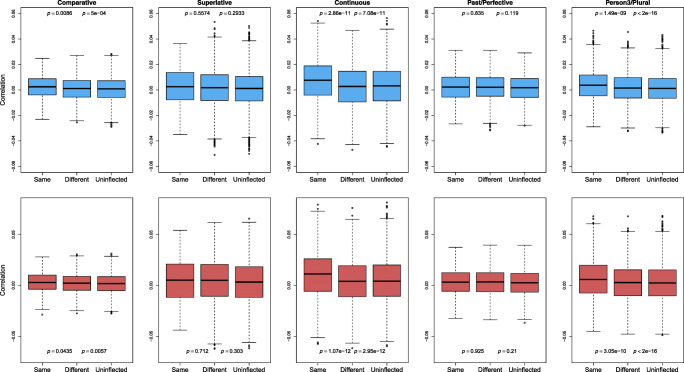
Boxplots of inflected pseudowords’ correlations with other pseudowords of the same, and of different inflectional categories, and with uninflected pseudowords. The *upper panel* presents the correlations calculated based on the direct route, and the *lower panel* presents those calculated based on the indirect route. The *p* values in the graphs are taken from regression models in which correlations are predicted by different inflectional categories. The first *p* value is for the comparison between Same and Different categories, and the second one is for that between Same and Uninflected categories

To gauge semantic similarities between inflected and uninflected pseudowords, we calculated, for each pseudoword, its correlations with all the other pseudowords.^9^ Figure 5 presents boxplots, for each of the five inflectional categories that we distinguish, that visualize the distributions of correlations for three subsets of pseudowords: pseudowords belonging to the same inflectional category (Same), pseudowords belonging to a different inflectional category (Different), and pseudowords that are uninflected (Uninflected). Within each panel, two *p* values are listed, the first referring to the Same and Different subsets, and the second to the Same and Uninflected subsets. These *p* values are taken from regression models in which the Same subgroup is the reference level of a factor type (using treatment coding), and pertain to standard *t* tests evaluating the presence of significant differences with the reference level.

As can be seen, significant contrasts are detected for pseudowords with the inflectional functions of comparative, continuous, and person3/plural, with respect to both Different and Uninflected subsets. The absence of differentiation for the superlative is likely due to the small number of superlative forms (110): although the superlative forms are technically ambiguous, it seems to us that a form such as is most plausibly a superlative. The past/perfective forms are systematically ambiguous, and hence the absence of semantic clustering is perhaps unsurprising. The emergence of some clustering for person3/plural might be due to the shared underlying dimension of number: singular for verbs and plural for nouns. Possibly the semantic vectors have picked up this commonality with respect to number.

**Fig. 6:**
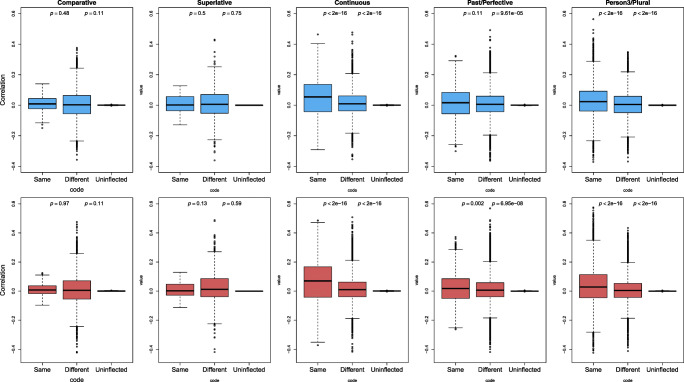
Boxplots of inflected pseudowords’ correlations with real words of the same, different inflectional categories, and with uninflected words. The *upper panel* presents the correlations calculated based on the direct route, and the *lower panel* presents those calculated based on the indirect route. The *p* values in the graphs are taken from regression models in which correlations are predicted by different inflectional categories. The first *p* value is for the comparison between Same and Different categories, and the second one is for that between Same and Uninflected categories

We further expected that the same inflectional relation should be visible not only within pseudowords, but between pseudowords and real words as well. Thus, inflected pseudowords would be closer to words with the corresponding inflectional functions. The boxplots in Fig. 6 present the distributions of inflected pseudowords’ correlations with inflected and uninflected real words. The pattern of higher semantic similarity between pseudowords and words of the same inflectional functions is clearly seen in continuous, past/perfective, and person3/plural (the third to fifth columns). For comparative and superlative, higher correlations within the same inflectional categories were however not observed (the first and second columns). The small number of comparatives and superlatives in the dataset of real words (113 and 95 out of 12,175 inflected words), hence reflecting a lack of power, could be the reason for the absence of clear difference here. Taken together, the present results demonstrate that the semantics of inflectional functions do emerge to some extent for the pseudowords, provided that the pertinent inflectional functions are well enough attested in the training and test data.

### Pseudoword duration

**Fig. 7:**
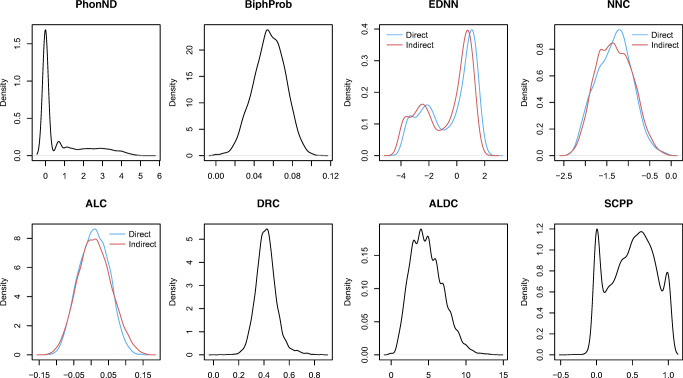
The density plots of transformed predictors in the present study. For EDNN, NNC, and ALC, which can be derived from the direct and indirect routes, two distributions are plotted

Predictors with skewed distributions were transformed.^10^ The distributions of the transformed predictors are presented in Fig. 7. The pairwise correlations between all the predictors are presented in Table 2. We note that for all the predictors, pseudowords significantly differ from words, according to two-sample Wilcoxon tests (for all predictors, *p* < 0.00001). In general, the LDL measures show that pseudowords are less “word-like”. Pseudowords tend to be more distant from the nearest word neighbor (larger EDNN), they tend to have larger angles with the nearest word neighbor (smaller NNC), they are located in a sparser semantic neighborhood (smaller ALC), their predicted pronunciations tend to deviate more from the targeted pronunciations, resulting in larger ALDC and smaller SCPP^11^.

**Table 2:** Pairwise Pearson correlations between predictors

	PhonND	BiphProb	EDNN_1_	NNC_1_	ALC_1_	EDNN_2_	NNC_2_	ALC_2_	DRC	ALDC	SCPP
PhonND	1	-0.13	**-0.41**	0.21	0.11	**-0.41**	0.20	0.14	0.14	**-0.62**	0.10
BiphProb	-0.13	1	0.11	-0.05	-0.01	0.11	-0.03	-0.02	-0.04	0.11	0.15
EDNN_1_	**-0.41**	0.11	1	**-0.45**	-0.28	1	**-0.46**	-0.33	-0.33	**0.42**	0.00
NNC_1_	0.21	-0.05	**-0.45**	1	**0.57**	**-0.45**	**0.62**	**0.49**	0.33	-0.18	0.01
ALC_1_	0.11	-0.01	-0.28	**0.57**	1	-0.28	**0.54**	**0.87**	0.19	-0.09	0.01
EDNN_2_	**-0.41**	0.11	1	**-0.45**	-0.28	1	**-0.46**	-0.33	-0.32	**0.42**	0.00
NNC_2_	0.20	-0.03	**-0.46**	**0.62**	**0.54**	**-0.46**	1	**0.68**	0.38	-0.17	0.00
ALC_2_	0.14	-0.02	-0.33	**0.49**	**0.87**	-0.33	**0.68**	1	0.21	-0.11	0.01
DRC	0.14	-0.04	-0.33	0.33	0.19	-0.32	0.38	0.21	1	-0.13	-0.01
ALDC	**-0.62**	0.11	**0.42**	-0.18	-0.09	**0.42**	-0.17	-0.11	-0.13	1	-0.38
SCPP	0.10	0.15	0.00	0.01	0.01	0.00	0.00	0.01	-0.01	-0.38	1

Subscripts 1 and 2 indicate measures derived from the direct and indirect route respectively. Absolute correlations between different predictors greater than 0.4 are highlighted in *bold*

We first fitted a generalized additive model (GAM) to square-root transformed pseudoword duration with as predictors PhonND and BiphProb, the two phonological form measures that have been the focus of previous pseudoword studies (cf. Vitevitch et al., 1997; Vitevitch and Luce, 1999). Given the semantic difference between inflected and uninflected pseudowords (Section “Pseudoword morphology”), a binary variable that indicates whether a pseudoword is inflected or not (IsInfl) was included in the model as well.^12^ A summary of this model is provided in Table 3.

**Table 3:** GAM fitted to square-root transformed pseudoword duration with the phonological measures as predictors. s: thin plate regression spline smooth

A. Parametric coefficients	Estimate	Std. Error	t value	*p* value
Intercept	24.0155	0.0287	835.68	< 0.0001
IsInfl:TRUE	0.3693	0.0447	8.263	< 0.0001
B. Smooth terms	edf	Ref.df	F-value	*p* value
s(PhonND)	5.365	6.260	937.55	< 0.0001
s(BiphProb)	1.001	1.001	88.36	< 0.0001

-ML = 20876, *R*^2^(adj) = 0.396

**Fig. 8:**
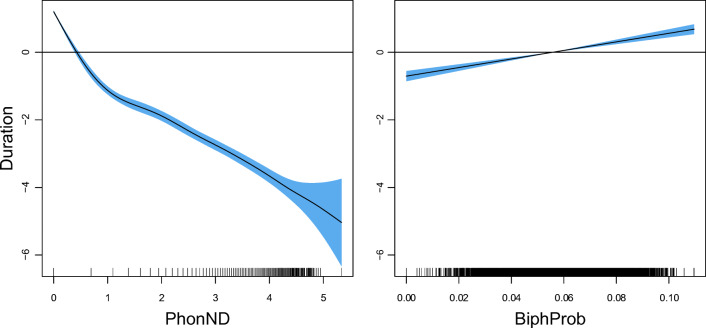
The partial effects of the phonological measures on square-root transformed pseudoword duration

Both phonological measures are significantly predictive for pseudoword duration. For PhonND, the effect of which is visualized in the left panel of Fig. 8, durations are shorter with increasing PhonND. The effect of BiphProb (Fig. 8, right panel), on the other hand, is linear but with a substantially reduced effect size and opposite sign: pseudoword duration is longer when phonotactic probabilities are higher. The effects of PhonND and BiphProb are in general in line with the findings of previous studies. Gahl, Yao, and Johnson (2012), for example, examined the effect of PhonND on word duration in spontaneous speech. Their hypothesis, which is based on the two-step interactive activation model of lexical access of Dell (1986) and Dell and Gordon (2003), is that denser phonological neighborhood is associated with shorter duration. As argued in Dell and Gordon (2003), phonological neighbors collectively generate support for a target via feedback from phonological segments, but do not constitute target competitors: The target gets strong semantic support during early (semantically driven) stages of lexical selection, which the phonological neighbors lack. Such a shortening effect of PhonND on duration was confirmed by their study for words, as well as the present study for pseudowords.^13^ With regards to BiphProb, although the investigation of its effect in Gahl et al., (2012) is less direct, due to its high correlation with PhonND (*r* = 0.62), they reported a marginal trend for larger BiphProb (after residualization on PhonND) leading to longer duration. For the pseudowords examined here, the two measures are not correlated (*r* = − 0.13), possibly due to a wider range of pseudoword lengths and syllable structures in the current dataset. Nevertheless, we similarly observed a lengthening effect of BiphProb on duration.

A second GAM was fitted to pseudoword duration with the LDL measures as predictors. However, as shown in Table 2, these predictors are highly correlated with one another. For example, for EDNN and NNC, *r* = − 0.45 for the direct route and − 0.46 for the indirect route. For NNC and ALC, *r* = 0.57 and 0.68 respectively. Moreover, the two-route correlations for these three measures are as high as 1, 0.62, and 0.87. Given the high correlations among the LDL predictors (*κ* = 159.91), especially those derived from the comprehension model, we performed a principal component analysis (PCA) regularization on the subset of semantic measures (EDNN_1_, NNC_1_, ALC_1_, EDNN_2_, NNC_2_, ALC_2_, DRC)^14^. The newly derived orthogonal dimensions (PCs) were predictive for acoustic durations to a substantial extent. However, because of the complicated inter-relations among these measures, these PCs were uninterpretable. Subsequent analyses revealed that the results of regression analyses stayed qualitatively the same when we used only measures from one of the two routes, unsurprisingly given the high similarity of the semantic vectors estimated by the two routes (cf. Fig. 7). Although overall model fit is slightly better with the omnibus analysis, for being able to interpret the effects of these semantic measures, we opted to use only predictors derived from the direct route (i.e., EDNN_1_, NNC_1_, ALC_1_), and to rely on DRC to inform us about how similar the predictions generated by the two routes are.

**Fig. 9:**
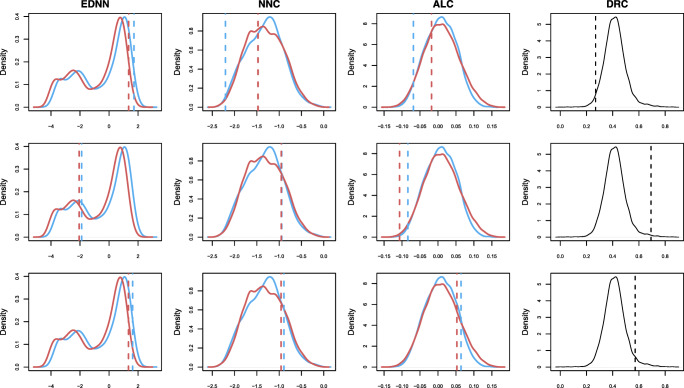
The values of the four semantic measures for three pseudowords: (top), (mid), and (bottom), which have high PC1, PC2, and PC3 values, respectively. The *dashed lines* highlight the position of the three words with respect to distributions. The *blue lines* are for the direct route, while the *red lines* are for the indirect route. The *upper left panel* shows that has a high value for EDNN, and hence, given Table 4, has a high value on PC1

Even with only measures from the direct route, the model is still beset with high collinearity (*κ* = 139.73). We thus again performed PCA on EDNN, NNC, ALC (of the direct route), and DRC. The PCA loadings for these four measures are shown in Table 4. To illustrate the relation between PCs and the original measures, Fig. 9 presents the original values of the four measures of three pseudowords: , and , which have high PC1, PC2, and PC3 values, respectively. Table 4 shows that PC1 contrasts EDNN with the other three measures. When PC1 is large (Fig. 9, top panels, illustrates this for the pseudoword ), a pseudoword is distant from other words (high EDNN), not semantically similar to any word (low NNC), and lands in a sparse neighborhood (low ALC). At the same time, the semantic predictions of the two routes are dissimilar (low DRC). In Fig. 9 (top center panels), this is illustrated by the separation of the dashed vertical lines representing the direct and the indirect routes. In other words, a pseudoword with a high value of PC1 has ended up in a semantically isolated region without many surrounding word neighbors, although exactly where the pseudoword is located seems to be route-dependent.

**Table 4:** PCA loadings for the four semantic measures

	PC1	PC2	PC3
EDNN	0.49	-0.29	0.78
NNC	-0.59	-0.24	0.03
ALC	-0.50	-0.59	0.28
DRC	-0.41	0.72	0.56

PC2, on the other hand, contrasts DRC primarily with ALC. A pseudoword with a large PC2 value (Fig. 9, mid panels, illustrates this for the pseudoword ) is again in a sparse neighborhood (low ALC), but both the direct and indirect routes produce similar semantic vectors (high DRC). Both routes predict a relatively sparse semantic neighborhood for the pseudoword, but it is still likely to have a close word neighbor nearby.

As for PC3, this orthogonalized dimension aligns EDNN with DRC. Similar to PC1, larger values of PC3 (Fig. 9, bottom panels, illustrates this for the pseudoword ) again indicate that a pseudoword is very distant from its nearest neighbor (high EDNN). However, unlike PC1, the meanings generated by the two routes are similar (high DRC). This suggests that the pseudoword again lands in a far-away semantic space, but both routes yield very similar predictions.

The three PCs together account for 90% of the variance in the semantic measures. With the three PCs, the condition number for all the LDL predictors together (PC1, PC2, PC3, and the two production measures ALDC and SCPP) is reduced to an acceptable value (*κ* = 12.20). In our analysis, we included one factorial predictor, IsInfl, a binary variable indicating the presence of a potential inflectional ending, that we also used in the analysis of standard phonological measures (see Table 3).

In the following analyses, we included not only the production measures but also the comprehension measures for predicting the acoustic durations of pseudowords. The reasons for including comprehension measures are two-fold. First, the speaker was requested to name psedudowords from IPA transcriptions multiple times. Given that he heard himself saying the pseudowords, a comprehension component is therefore inevitably involved in his production. In addition, the effect of phonology on reading has been well documented (Wong & Chen, 1999; Newman, Jared, & Haigh, 2012; Jared, Ashby, Agauas, & Levy, 2016; Jared & Bainbridge, 2017), and even for silent reading, readers usually hear their “inner voice” (Perrone-Bertolotti et al., 2012). Our model explicitly takes these complex interactions between comprehension and production into account in a principled way. Second, the LDL model assumes that speech production involves internal comprehension loop, implementing “synthesis-by-analysis”; for extensive discussion and motivation, see Baayen et al., (2018a). The SCPP measure gauges the consequences of the internal comprehension process guiding production (see, Hickok, 2014, for neuro-cognitive evidence for interaction between the comprehension and the production systems).

**Fig. 10:**
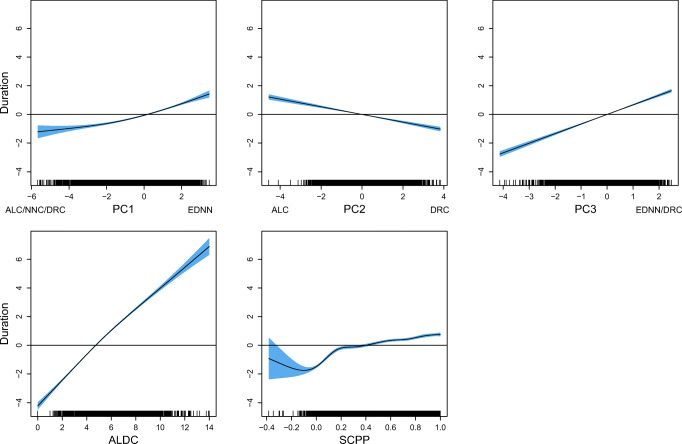
The partial effects of the LDL measures on square-root pseudoword duration. For each of the PCs (*upper panels*), measures with positive loadings on a PC are indicated at the higher end of the scale, whereas measures with negative loadings are indicated at the lower end

The model summary is presented in Table 5. All LDL measures are reliable predictors for pseudoword duration. We first consider the two measures derived from the production model, the partial effects of which are shown in the lower panels of Fig. 10. For ALDC, the positive effect on pseudoword duration is nearly linear with a large effect size. As the candidate forms generated by the model (on the basis of the internal conceptualization of a pseudoword’s meaning) are more distant from the intended pronunciation of a pseudoword, its duration tends to be longer. With respect to SCPP, which gauges the semantic correlation between the form realized by the speaker and the best candidate form given its perceived semantics, we again observe a positive, but substantially attenuated and non-linear effect on duration. For real words, two studies, Kuperman, Pluymaekers, Ernestus, and Baayen (2006) and Cohen (2014), have reported acoustic strengthening in conditions with reduced uncertainty. We interpret the effects of ALDC and SCPP in the same way. When the predicted pseudoword form is not confusable with other forms, and when there is little discrepancy between the semantics projected by the spoken form and the form generated from its semantics, durations increase.

**Table 5:** GAM fitted to square-root transformed pseudoword duration with the LDL measures as predictors. s: thin plate regression spline smooth

A. Parametric coefficients	Estimate	Std. Error	t-value	*p* value
Intercept	24.0092	0.0238	1010.7	< 0.0001
IsInfl:TRUE	0.3844	0.0375	10.26	< 0.0001
B. Smooth terms	edf	Ref.df	F-value	*p* value
s(PC1)	3.065	3.889	161.6	< 0.0001
s(PC2)	1.001	1.001	173.0	< 0.0001
s(PC3)	1.000	1.000	792.8	< 0.0001
s(ALDC)	3.279	4.150	1495.6	< 0.0001
s(SCPP)	7.656	8.555	141.7	< 0.0001

-ML = 18963, *R*^2^(adj) = 0.596

The upper panels of Fig. 10 visualize the effects of these three PCs. For PC1, larger values give rise to longer duration, indicating that when a pseudoword is semantically at a greater distance from its word neighbors and semantically not very confusable with real words, its duration tends to be lengthened. PC1 may be capturing pseudowords that stand out semantically as pseudowords, similar to the way in which a novel taste can stand out as distinct from previously experienced tastes. This effect mirrors that of ALDC, in that the general trends have positive slope.

Larger PC2 values, on the contrary, lead to shorter duration. This depicts a situation where the semantics estimated by the two routes converge, but where the pseudoword is in a sparse neighborhood. PC3 shows an upward trend similar to PC1, but with a stronger effect size. When PC3 is larger, it is again the case that the semantics of the two routes converge, but now the pseudoword is farther away from its nearest neighbor. Apparently, PC2 and PC3 are gauging two opposite orthogonal effects in the presence of route convergence, that differentiate between semantic density and semantic distance. It thus appears that on the one hand, route convergence can be beneficial, in the sense that a consistent interpretation is obtained. This effect is conditioned by large semantic distance of the pseudoword from other words. In this case, PC3 affords durational lengthening. On the other hand, when in a sparse neighborhood, the similarity of two meanings would increase entropy in the system, leading to durational shortening (PC2).

Compared to the GAM with the standard phonological measures as predictors, the model fit of the model with the LDL measures is substantially better, indicated by a decrease of ML scores by 1913. However, if we add the two standard phonological measures as additional predictors to the latter model, model fit still improves (ML scores decrease by 296.71), but the effect sizes of the two phonological measures are substantially attenuated. While larger PhonND again leads to shorter duration (similar to Fig. 8, left panel), the effect of BiphProb levels off with a tiny effect size. It thus seems that part of the PhonND effect and almost the entire BiphProb effect have been captured by the LDL measures. We will return to the theoretical consequences of the remaining effect of phonological neighborhood density in the general discussion.

### Pseudoword reaction time

The original data of pseudoword RTs provided by the MALD database were measured from stimulus onset. Given that this RT measure includes duration, it is unsurprising that duration and RT are well correlated (*r* = 0.37). In fact, duration is the dominant predictor for RT. To avoid issues of collinearity, specifically arising here because duration itself is co-determined by the present predictors, we subtracted duration from reaction time, focusing on RT measured from pseudoword offset. About 1.2% of the offset RTs are negative, indicating early responses before offset. These tokens were excluded from the analyses.

**Fig. 11:**
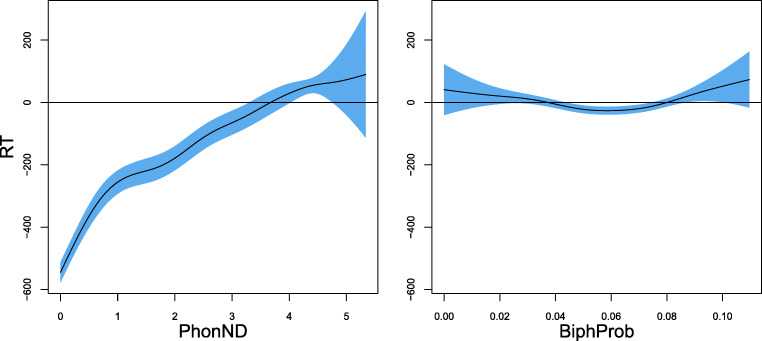
The partial effects of the phonological measures on Box-Cox transformed pseudoword RT

Due to the very positively skewed distribution of RTs, we applied a Box-Cox transformation to RTs with a *λ* value of 0.26. As for the duration analyses, we first fitted a GAMM to RTs with the two phonological measures as predictors. The indicator variable for inflection, IsInfl, along with by-subject and by-pseudoword random intercepts, were included as well. A model summary is provided in Table 6. Both phonological effects are significant, but the roughly linear effect of PhonND is much stronger than the small U-shaped effect of BiphProb (see Fig. 11). This pattern of results fits well with the finding of Vitevitch and Luce (1999) that the perception of pseudowords in a lexical decision task is primarily determined by the effect of PhonND. According to Vitevitch and Luce, because the task of lexical decision requires listeners to explicitly distinguish pseudowords from words, pseudowords with more phonological neighbors, which induce more severe lexical competition, are thus more difficult to reject.

**Table 6:** GAMM fitted to pseudoword Box-Cox transformed RT with the phonological measures as predictors. s: thin plate regression spline smooth

A. Parametric coefficients	Estimate	Std. Error	t-value	*p* value
Intercept	5231.12	29.34	178.30	< 0.0001
IsInfl:TRUE	-15.487	7.21	-2.15	< 0.05
B. Smooth terms	edf	Ref.df	F-value	*p* value
s(PhonND)	5.495	5.804	550.410	< 0.0001
s(BiphProb)	3.579	3.892	5.118	< 0.001
s(Subject)	227.98	230	176.177	< 0.0001
s(Pseudoword)	6218.92	9569	7.461	< 0.001

fREML = 159885, *R*^2^(adj) = 0.324

**Fig. 12:**
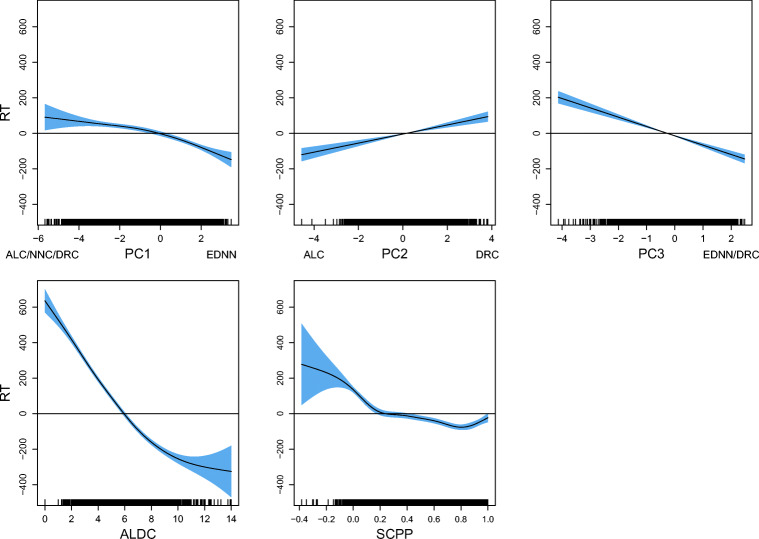
The partial effects of the LDL measures on Box-Cox transformed pseudoword RT. For each of the PCs (*upper panels*), measures with positive loadings on a PC are indicated at the higher end of the scale, whereas measures with negative loadings are indicated at the lower end

A second GAMM was fitted to predict RTs from the same LDL measures that we used for modeling acoustic durations. A summary of the model is presented in Table 7. For the three PCs (Fig. 12, upper panels), the effects are mostly linear or close to linear. PC1 has a negative effect on RTs, with larger PC1 values leading to shorter RTs. Thus, response times are reduced when a pseudoword is semantically distinct, i.e., at greater distance from its nearest semantic neighbors. The effect of PC2, on the other hand, exhibits an upward trend, with RTs becoming longer as PC2 increases. Finally for PC3, as for PC1, a downward trend is present.

**Table 7:** GAMM fitted to Box-Cox transformed pseudoword RT with the LDL measures as predictors. s: thin plate regression spline smooth

A. Parametric coefficients	Estimate	Std. Error	t-value	*p* value
Intercept	4731.07	25.85	183.0	< 0.0001
IsInfl:TRUE	-23.21	7.03	-3.3	< 0.001
B. Smooth terms	edf	Ref.df	F-value	*p* value
s(PC1)	2.770	3.051	44.643	< 0.0001
s(PC2)	1.013	1.018	44.579	< 0.0001
s(PC3)	1.002	1.002	139.552	< 0.0001
s(ALDC)	4.643	5.046	438.29	< 0.0001
s(SCPP)	6.135	6.559	51.12	< 0.0001
s(Subject)	227.00	230.00	170.43	< 0.0001
s(Pseudoword)	5870.70	9566	6.368	< 0.0001

fREML = 158846, *R*^2^(adj) = 0.325

The two measures from the production part of the model (Fig. 12, lower panels) also account for the variance in the RTs to a substantial extent. ALDC has a robust effect on RTs, with larger ALDC values leading to shorter RTs. As to SCPP, its effect, similar to that of ALDC, exhibits a downward trend, but it is more non-linear and its effect size is smaller. Generally, the effects on RTs are near mirror images of the effects on duration. Apparently, the same factors driving durational lengthening for the speaker allow listeners, after stimulus offset, to quickly reject pseudowords as real words.

With the LDL measures, model fit again significantly improved compared to the model with standard phonological predictors, with fREML scores decreasing by 1039 units. Although the inclusion of PhonND and BiphProb into the current model still improved model fit (Δ fREML = 351.2 units), their effect sizes were substantially reduced.

## Discussion

**Fig. 13:**
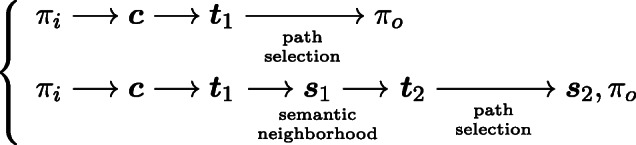
LDL processing stages. *π*_*i*_ represents spatially or temporally ordered visual or auditory input. ***c*** represents a modality-specific cue vector, ***t*** denotes a triphone vector, ***s*** is a semantic vector, and *π*_*o*_ denotes an ordered sequence of triphones that is the input for articulation. The *upper flow* denotes naming bypassing semantics, the *lower flow* represents naming, comprehension, and production with semantics. Semantic measures (EDNN, NNC, ALC) quantify the semantic neighborhood of ***s***_1_. The ALDC measure quantifies path selection, and the SCPP measure is the correlation of ***s***_1_ and ***s***_2_. The direct route in comprehension bypasses ***t***_1_

In this study, we made use of the LDL framework to model the production and perception of auditory pseudowords. The relevant processing stages, summarized in Fig. 13, can be grouped into two pathways (cf. Coltheart, Curtis, Atkins, & Haller, 1993; 2001). The upper pathway does not involve semantics. The visual input (*π*_*i*_), which is a sequence of letters ordered in space, is transformed into an unordered visual cue vector (here of IPA triphones) ***c***, which is then mapped onto a triphone vector ***t***_1_, representing the internal auditory image of the pseudoword. Because there is a one-to-one mapping between the IPA transcriptions read by the speaker and the triphones in ***t***_1_, the path selection algorithm operating on ***t***_1_ performs nearly error-free: when the threshold for thinning the triphone graph is set close to 1, there typically is only one candidate path. In this way, we model that the speaker nearly always produce the targeted pseudowords from print.

What is central to the current study is the lower processing path depicted in Fig. 13, which does involve semantics, and which we assume to proceed in parallel with the upper path. For production driven by internal conceptualization, the production process starts at the semantic vector ***s***_1_. This vector is mapped onto the triphone vector ***t***_2_, from which our path selection algorithm selects the best path. This algorithm implements synthesis-by-analysis for selecting the best path. The best path is that path for which the corresponding predicted semantic vector (***s***_2_) is closest to the targeted semantic vector (***s***_1_). This path (*π*_*o*_) in turn drives articulation.

For the naming of real words, the triphone vector ***t***_1_ is assumed to be mapped into the semantic space. This mapping is independently required for not only production but also comprehension with the indirect route. The resulting semantic vector ***s***_1_ then passes through the production process as described above. For real words, the mappings both onto ***s***_1_ and subsequently onto ***t***_2_ are well learned, such that the input for articulation (*π*_*o*_) will typically match the output from the direct naming route (the upper path in Fig. 13). However, for pseudowords, this is not necessarily the case: the predicted semantic vector ***s***_1_ typically lands in a region of semantic space where it is not that close to the semantic vectors of real words. Furthermore, in the synthesis-by-analysis process, the paths that are predicted from this semantic vector in turn predict semantic vectors that are not very close to the semantic vector ***s***_1_ that is to be realized in speech. This holds even for the semantic vector ***s***_2_, which is the most similar to the targeted semantic vector ***s***_1_.

With respect to auditory comprehension, for which semantics is always involved, only the lower path is at play. The temporally ordered auditory input is transformed into an unordered FBS feature vector ***c***. Depending on which route is taken, ***c*** is mapped onto the semantic vector ***s***_1_ either directly or indirectly via the triphone vector ***t***_1_. This is usually the end stage of processing in classical models of auditory comprehension. However, in our model, the comprehension and production systems interact such that the semantic vector ***s***_1_ is subsequently mapped onto a triphone vector ***t***_2_, which in turn leads to the activation of the semantic vector ***s***_2_. For real words, the processing steps are well learned, and ***s***_2_ will be similar or identical to ***s***_1_. Thus little uncertainty arises in the system. By contrast, pseudowords give rise to much more uncertainty. The semantic vectors ***s***_1_ and ***s***_2_ will be typically much less similar than in the case of real words. As a consequence, the measures for semantic neighborhood and path selection are much more informative for pseudowords as compared to words.

Thus, it is specifically for pseudowords that measures of semantic neighborhood, as well as measures of path selection uncertainty can be predictive for speech production, as gauged by acoustic durations, and for auditory comprehension, as gauged by auditory lexical decision latencies.

A further consideration is that pseudowords do not have the rich semantics that characterizes real words. They land in an area of semantic space that does not link up to discriminated experiences of the world. This explains why upon reading or hearing a pseudoword, there is no clear semantic percept that reaches awareness. However, subliminal semantic processing does take place, as evidenced by the predictivity of our semantic and production measures. We suspect that for real words the clarity of the semantic percept and its link to real world knowledge may largely mask the effect of the resonance between the production and comprehension systems. If this conjecture can be shown to be correct, the implication is that pseudowords are especially felicitous tool for investigating this resonance experimentally.

The results obtained in this study have several implications for the theory of the mental lexicon. First, instead of taking production and comprehension to be mechanical processes that are independent of each other, LDL implements a model in which the two subsystems resonate, and in which forms and meanings emerge out of the ‘negotiations’ between these subsystems.

Second, pseudowords clearly are not semantically empty. In fact, this conclusion is not new in linguistics. The meaning of sublexical units has been extensively discussed in the literature of sound symbolism (e.g., Köhler, 1929; Sapir, 1929; Westbury, 2005; Maurer et al., 2006; Imai & Kita, 2014; Westbury et al., 2018), providing evidence that pseudowords are to some extent interpreted. In the domain of computational modeling research, however, the possibility that pseudowords might have their own specific semantics has not been explored. The focus has been on distinguishing words from pseudowords either on the basis of the properties of activation patterns (e.g., McClelland & Rumelhart, 1981; Grainger & Jacobs, 1996; or on the basis of a hypothesized general nonword node or representation (Norris & McQueen, 2008).

Third, the mappings learned between form and meaning in LDL enable morphological effects to emerge straightforwardly. With respect to the pseudowords in our data which potentially carry inflectional exponents, we found that they are to some extent semantically distinct from those pseudowords that do not have inflectional exponents. Furthermore, pseudowords are semantically more similar to words and pseudowords carrying the same inflectional exponents, as compared to words and pseudowords with different inflectional exponents. This suggests that these pseudowords are projected into semantic subspaces populated with semantic vectors of inflectionally related words. We note that for inflected pseudowords, the semantics of the pseudoword stems still remain relevant—otherwise, the semantic vectors of inflected pseudowords would be identical for all pseudowords with the same exponent, contrary to fact. From this, we conclude that auditory comprehension of morphologically complex words is algorithmically possible without ever having to parse the speech signal into discrete pre-existing constituents. In other words, our results demonstrate that pseudowords can be processed morphologically without mediation by morpheme units. This strengthens the case, made by Baayen et al., (2019), that real words are also produced and understood without the involvement of morpheme units.

Fourth, the semantic and formal neighborhood structures of pseudowords co-determine pseudoword processing. To gauge semantic neighborhood properties, we calculated both the Euclidean distance and the angles between semantic vectors (EDNN, NNC, ALC). As to form neighborhood, here we calculated the average distance between the targeted pronunciation form and the candidate forms generated by the path selection algorithm (ALDC). While these neighborhood effects receive robust statistical support, they however require further discussion in the context of the LDL framework. This framework, as laid out in Fig. 1, comprises a set of inter-connected networks (implemented with linear mappings). Exemplars leave traces in these networks, but are not stored as such. They exert their influence during learning, but do not have an independent ontological status. Although we define matrices such as ***C***_*a*_, ***C***_*o*_, ***S***, and ***T***_*a*_, the rows of which specify properties of exemplars, these matrices also do not have independent ontological status. It is for computational convenience that in the present study we use linear algebra to estimate the mappings. In theory, learning is incremental. Incremental learning of linear mappings is possible with the update rule of Widrow and Hoff (1960), but for the relatively small training set of the MALD real words, however, the estimation method using linear algebra is both efficient and precise. Conceptually, LDL is an exemplar-free model.

This raises the question of how the measures derived from the model, which make reference to neighbors, should be understood. For semantic neighbors (on which the EDNN, NNC, ALC, and DRC measures are based), our tentative answer to this question builds on the hypothesis that a semantic vector is not a goal in itself, but rather that a semantic vector makes further contact with words’ affordances (Gibson, 1977). Neighbor vectors thus provide glimpses of the kind of affordances that a pseudoword is co-activating to some extent. By way of example, consider a blend such as *brunch*, and suppose that this blend has not been encountered before. Suppose its semantic vector is in the proximity of the semantic vectors of *breakfast* and *lunch*. The vectors $\overrightarrow {\text {breakfast}}$ and $\overrightarrow {\text {lunch}}$ make contact with typical meals and the time of day for which these meals are scheduled. By considering the proximity of $\overrightarrow {\text {brunch}}$ to $\overrightarrow {\text {lunch}}$ and $\overrightarrow {\text {breakfast}}$, we are in fact assessing to what extent the affordances provided by the neighbors make sense. For *brunch*, a blend that was created in England in the late 19th century, a sensible merger of affordances has led to the interpretation of a combined breakfast and lunch, eaten in the late morning or early afternoon (see Gries, 2004, for discussion of what makes blends successful). Thus, if a pseudoword vector lands in a sparsely populated area in semantic space, little contact is made with existing affordances, and the affordances of the distant neighbors will be more diverse and more difficult to make sense of. Such a state of affairs leads to fast rejection times in auditory lexical decision and to longer acoustic durations in speech production.

Form neighbors come into play with the ALDC measure. This measure evaluates the similarity of the pseudoword form produced with the neighbors projected from the pseudoword’s semantic vector. We do not take these form neighbors to be competing lexical entries. We think of them more as alternative realizations that are also supported to some extent by the semantics of the pseudoword. These neighbors are rather similar to pronunciation variants or speech errors, such as English *blazed* being realized with final instead of , or *mouths* being realized with as coda instead of . Thus, the ALDC measure provides a means for assessing the uncertainty about the proper form of a pseudoword given its meaning. Greater values of ALDC are indicative of neighbors being less similar. Reduced neighbor similarity implies decreased lexical uncertainty, and this in turn affords shorter reaction times and longer durations.

Although a regression model with LDL predictors provides a more precise fit to the reaction times and acoustic durations compared to a regression model with classical measures of lexical similarity, we observed above that adding the neighborhood density measure as an additional predictor to the LDL regression model results in a further improvement in goodness of fit. This result suggests that we are still missing out on an important aspect of lexical similarity. Our current hypothesis is that this is due to the LDL model being deterministic rather than stochastic. Model input is represented by vectors of units that are either on or off. In real biological systems, what we represent with a unit for the presence or absence of a feature is in reality an assembly of cells each of which is more or less likely to fire given some input. Furthermore, synaptic connectivity has also been observed to be stochastic (see Kappel et al., 2015, 2017, for error-driven learning models with stochastic connection weights). To make the LDL model more realistic, a deterministic form representation will have to be replaced by a distribution of form representations, and a deterministic linear mapping will likewise require replacement by a stochastic linear mapping. The result of a form-to-meaning mapping will thus be a distribution of semantic vectors. Our hypothesis is that neighborhood similarity effects are intrinsic to stochastic systems, and that ‘neighbors’ are actually less probable network states in a superposition of network states in which the targeted state is (ideally) the most probable one. We leave the formalization of this intuition, which could be pursued within a Bayesian framework (see, e.g., Kappel et al., 2015) or using methods from quantum physics (for an implementation of an exemplar model using quantum computing, see Skousen, 2000) to future research.

Finally, this study is not the first to demonstrate that the processing of pseudowords may involve semantics (see, e.g., Cassani et al., 2019). In the context of the present study, the investigation by Hendrix and Sun (2020) of the processing of pseudowords in the visual lexical decision task is of special interest. Similar to our study, they also generated semantic vectors for pseudowords, though using a different computational method (fastText, Bojanowski, Grave, Joulin, & Mikolov, 2017), and calculated the angles between the semantic vectors of pseudowords and those of words. They found that pseudowords that are semantically more dissimilar to real words are rejected more quickly, which is consistent with our findings for auditory pseudowords. In addition to pseudoword semantics, they further observed an effect of pseudoword frequency. Interestingly, nearly all of the pseudowords they examined (a subset from the British Lexicon Project, Keuleers et al., 2012) have a non-zero frequency count according to a Google search. These pseudoword frequency counts appear to have a non-negligible effect on visual pseudoword processing, with greater frequencies predicting longer rejection times. The potential effect of frequency on auditory pseudoword processing is an interesting topic worth further pursuit. However, given that orthographic information is not available for pseudowords in the MALD database, and given the amount of irregularity in the sound-spelling correspondence in English, it is unclear how to best estimate the frequency of auditory pseudowords. Nevertheless, in light of these accumulating results, it is now becoming clear that much more can be said about pseudoword processing than previously assumed, and that even more remains to be discovered.

### Electronic supplementary material

(PDF 283 KB)

## Figures and Tables

**Fig. 14:**
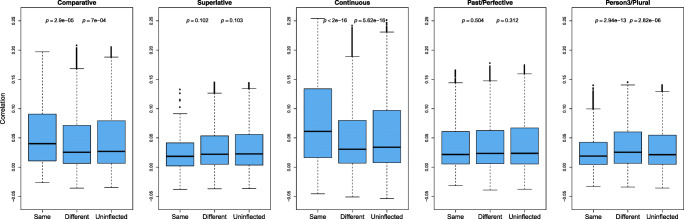
Boxplots of inflected pseudowords’ correlations with other pseudowords of the same, different inflectional categories, and with uninflected pseudowords. The semantic vectors of pseudowords are derived from the direct route, based on Word2Vec embeddings. The *p* values in the graphs are taken from regression models in which correlations are predicted by different inflectional categories. The first *p* value is for the comparison between Same and Different categories, and the second one is for that between Same and Uninflected categories

## Appendix: LDL with tweet-based Word2Vec embeddings

To test whether the above results are specific to the NDL-based semantic vectors that we used, a separate analysis was carried out by using a different algorithm for constructing semantic vectors, applied to a different language register. We downloaded the word embeddings from https://www.spinningbytes.com/resources/wordembeddings/ (Cieliebak, Deriu, Egger, & Uzdilli, 2017; Deriu et al., 2017). These embeddings are 200-dimension vectors, which were trained with Word2Vec on 200 million English Tweets. For the MALD words, 26,283 embeddings are available in this resource, resulting in a 26,283×200 semantic matrix ***S***. Note that inflectional homographs are not distinguished, and receive exactly the same embedding (that is, the verb *walks* and the noun *walks* are treated identically). Following the same procedures as described above, we built both a production and comprehension model. Again two estimated semantic vectors were derived for each pseudoword, one based on the direct route and the other based on the indirect route.

The first question is whether inflectional semantics can be captured by the networks trained with the Word2Vec-based embeddings. To address this issue, we again examined whether pseudowords labeled with a given inflectional function are semantically more similar to those with the same inflectional function, as compared to those with different inflectional functions or uninflected pseudowords. As highly similar patterns emerge for the two routes, Fig. 14 only presents boxplots for the correlations derived from predictions of the direct route. For comparative and continuous pseudowords, correlations with pseudowords of the same function are indeed higher than those of different functions, and higher also for uninflected pseudowords. However, for the other three inflectional functions, higher within-group correlations are not found. For person3/plural pseudowords, the effect is in the opposite direction: they are more correlated with, and thus semantically more similar to, the non-person3/plural inflected pseudowords and uninflected pseudowords. We conclude that from a morphological perspective, the Tweet-derived Word2Vec embeddings perform less well compared to the semantic vectors developed in Baayen et al., (2019) and that we used above.

We then calculated all the semantic and form measures exactly as defined above. As before, the distributions of the measures calculated from the two routes were very similar, details are available in the Supplementary Materials. A comparison of the measures obtained with Word2Vec and those obtained with NDL reveals very high correlation for the two distance measures (EDNN_1_ and EDNN_2_), see Table 8. The angle measures NNC and ALC, as well as the DRC measure, were less similar. With respect to the two production measures, whereas ALDC is very similar for the two embeddings, SCPP, on the other hand, show no correlation. In fact, the two production measures for the Word2Vec-based embeddings are not very reliable, since the production algorithm, even with a threshold for thinning the triphone graph lowered from 0.1 to 0.05, still failed to generate pronunciations for over half of the pseudowords (54%). This only happens for less than 10% of the pseudowords for the network using NDL semantic vectors. This is likely to be due to two factors. First, the dimensionality of the Word2Vec embeddings is only 200, whereas the dimensionality of the form space is 8601, almost two orders of magnitude greater. As a consequence, the precision of the mapping from semantics to forms must lose considerable precision. Second, the vectors in the semantic space set up by Word2Vec are much more similar to each other than is the case for the space set up by NDL (compare Fig. 5 and Fig. 14). Given the imprecision of the production measures, we considered only the semantic measures in the following regression analyses. Furthermore, as the measures derived from the two routes are so similar, we again used only the measures from the direct route in the statistical analysis.

**Table 8:** Spearman’s correlations between measures derived from networks using NDL and Word2Vec embeddings

EDNN_1_	EDNN_2_	NNC_1_	NNC_2_	ALC_1_	ALC_2_	DRC	ALDC	SCPP
0.94	0.94	0.38	0.39	0.24	0.29	0.20	0.75	-0.05

The high correlations among the semantic measures (e.g., *r*(EDNN,NNC) = − 0.81, *r*(EDNN,ALC) = − 0.84, *r*(NNC,ALC) = 0.89, and *r*(ALC,DRC) = 0.65) necessitated PCA orthogonalization; the first three PCs together accounted for 97.5% of the variance. The new PC1 had loadings that were very similar to the loadings of the NDL-based PC1, and hence has a very similar interpretation. The new PC2 had strong loadings for EDNN and DRC, and hence has a similar interpretation as the original PC3. The new PC3 showed strong loadings for EDNN and NNC, with high values for pseudowords for which the nearest neighbor is at a great distance but with small angle. In what follows, we only report the model fitted to acoustic durations, as the pattern of results for reaction times is the mirror image of that for the durations.

A summary of the GAM fitted to the acoustic durations is provided in Table 9. Clearly, the model with measures obtained with Word2Vec applied to Tweets is also predictive for pseudoword duration. Nevertheless, the model fit is slightly worse compared to that of the corresponding model with NDL-based measures as predictors (Table 10), i.e., using only the semantic measures and leaving out the production measures, with a difference of 18.42 AIC units. From this we conclude, first, that the NDL-based semantic vectors and the Word2Vec-based vectors generate similar predictions; and second, that the NDL-based measures do not perform worse, and may actually perform slightly better. We note here that Baayen et al., (2019) provide extensive validation of NDL-based semantic vectors, and that a comparison of the performance of NDL-based and Word2Vec-based modeling (Long, 2018) clarified that both methods have their advantages and disadvantages, a result which is in line with recent findings in computational linguistics (Melamud et al., 2016).

**Table 9:** GAM fitted to square-root transformed pseudoword duration with Word2Vec-based semantic measures as predictors. s: thin plate regression spline smooth

A. Parametric coefficients	Estimate	Std. Error	t-value	*p* value
Intercept	24.1695	0.0235	1031	< 0.0001
B. Smooth terms	edf	Ref.df	F-value	*p* value
s(PC1)	5.372	6.549	516.7	< 0.0001
s(PC2)	1.001	1.002	487.2	< 0.0001
s(PC3)	1.003	1.005	326.9	< 0.0001

-ML = 21540, *R*^2^(adj) = 0.306

**Table 10:** GAM fitted to square-root transformed pseudoword duration with NDL-based semantic measures as predictors. s: thin plate regression spline smooth

A. Parametric coefficients	Estimate	Std. Error	t-value	*p* value
Intercept	24.1695	0.0234	1032	< 0.0001
B. Smooth terms	edf	Ref.df	F-value	*p* value
s(PC1)	3.120	3.956	439.4	< 0.0001
s(PC2)	1.007	1.015	377.9	< 0.0001
s(PC3)	5.439	6.662	297.7	< 0.0001

-ML = 21533, *R*^2^(adj) = 0.308

## Footnotes

1 We note that the term “pseudoword” used here is equivalent to and referred to as “nonword” in many published studies.

2 Previous work has shown that, for form representations, letter trigrams and triphones capture the variability of neighboring orthographic and phonological information and usually work well not only for English (Milin, Feldman, Ramscar, Hendrix, & Baayen, 2017; Baayen et al., 2019) but also for many typologically different languages such as Latin (Baayen, Chuang, & Blevins, 2018a) and Estonian (Chuang, Lõo, Blevins, & Baayen, 2019). However, the optimal grain size of form representations can sometimes still be language-dependent. Bigrams, for example, have proven more suitable for Vietnamese due to its more restricted phonotactic constraints (Pham & Baayen, 2015). For modeling with lower-level visual features, see Linke, Broeker, Ramscar, and Baayen (2017).

3 See Melamud, McClosky, Patwardhan, and Bansal (2016) for the pros and cons of different techniques of deriving word embeddings for different NLP tasks.

4 An ongoing project in our lab is now developing a physical model of the vocal tract that generates speech directly based on articulatory gestural data (Sering, Stehwien, & Gao, 2019). This provides an alternative to triphone representation.

5 The support from the semantics for the triphones that are quantified in ***t***_*a*_ are very different from measures of phonotactic probability. These phonotactic probability measures are based on co-occurrence patterns in forms, and do not take semantics into account. Thus, mean biphone phonotactic probability is only very weakly correlated with LDL measures, as shown in Table 2.

6 We also calculated the average semantic correlation between all predicted forms and the target form. It is a measure very similar to SCPP (*r* = 0.81), and its effect on duration and reaction time is virtually indistinguishable from that of SCPP, and is therefore not discussed further.

7 Given the high variability of natural speech, it is very likely that there will be some novel FBS features in speech tokens of previously encountered words, in newly encountered out-of-vocabulary morphologically complex words, and for pseudowords. Nevertheless, for the present data, no less than 73% of the pseudowords have all their FBS features present. It is only for 2.3% of the pseudowords that more than five FBS features are missing in $\boldsymbol {C}_{a}^{(\text {pw})}$. Since missing FBS features have zero weights, they have no influence on the predictions.

8 Given that the labeling is only based on suffixes, we acknowledge the limitation of not taking irregular forms into consideration.

9 These correlations are necessarily small. The semantic vectors of morphologically complex pseudowords can be conceptualized as the sum of two vectors, namely the vector of the inflectional function ***i*** and that of the pseudoword stem ***k***. When comparing the semantic similarity of two complex pseudowords with the same exponent, the pseudoword semantic vectors (***p***_1_ and ***p***_2_) can be seen as noisy versions of the semantic vector ***i***: ***p***_1_ = ***i*** + ***k***_1_, ***p***_2_ = ***i*** + ***k***_2_. As ***k***_1_ and ***k***_2_ will tend to be much more orthogonal than is the case for real words, the semantic similarity between ***p***_1_ and ***p***_2_ is inevitably very low. The low correlations between pseudowords are consistent with the low activations reported by Harm and Seidenberg (2004) in their simulation study of pseudoword inflection.

10 PhonND, EDNN, and NNC were log-transformed. We took the square root of BiphProb, the cube of DRC, and the square of SCPP. The choice of transformation is motivated solely by the necessity to reduce outlier structure and stabilize subsequent regression modeling. Note that the distribution of PhonND is still very skewed after log transformation. This is because 63% of the MALD pseudowords has 0 phonological neighbors.

11 Comparisons of means between words and pseudowords, as well as the density plots of these measures for words, are provided in Supplementary Materials. For comparisons based on other phonological measures, see Tucker et al., (2018).

12 The number of phones does not differ significantly for inflected and uninflected words (*t*(9571) = − 0.79,*p* = 0.4). With regards to the number of syllables, although one might expect inflected pseudowords to have more syllables due to inflection such as , in fact in the MALD database, uninflected pseudowords have on average a somewhat larger mean syllable count than inflected pseudowords (2.7 vs. 2.3: *t*(9571) = 15.80,*p* < 0.0001). Hence, we conclude that IsInfl is not confounded with increased length in phones or syllables.

13 About 63% of the pseudowords do not have any phonological neighbors (cf. upper left panel, Fig. 7). Results remain largely the same when the pseudowords without any phonological neighbors are excluded from the dataset.

14 For strategies dealing with collinearity, see Tomaschek, Hendrix, and Baayen (2018).

## References

[CR1] Amenta S, Marelli M, Sulpizio S (2017). From sound to meaning: Phonology-to-semantics mapping in visual word recognition. Psychonomic bulletin & Review.

[CR2] Arnold, D. (2017). AcousticNDLCodeR: Coding sound files for use with NDL. R package version 1.0.1.

[CR3] Arnold D, Tomaschek F, Lopez F, Sering T, Baayen RH (2017). Words from spontaneous conversational speech can be recognized with human-like accuracy by an error-driven learning algorithm that discriminates between meanings straight from smart acoustic features, bypassing the phoneme as recognition unit. PLOS ONE.

[CR4] Baayen RH, Chuang Y-Y, Blevins JP (2018). Inflectional morphology with linear mappings. The Mental Lexicon.

[CR5] Baayen, R. H., Chuang, Y.-Y., & Heitmeier, M. (2018b). WpmWithLdl: Implementation of word and paradigm morphology with linear discriminative learning. R package version 1.0.

[CR6] Baayen RH, Chuang Y-Y, Shafaei-Bajestan E, Blevins J (2019). The discriminative lexicon: A unified computational model for the lexicon and lexical processing in comprehension and production grounded not in (de)composition but in linear discriminative learning. Complexity.

[CR7] Baayen RH, Dijkstra T, Schreuder R (1997). Singulars and plurals in Dutch: Evidence for a parallel dual route model. Journal of Memory and Language.

[CR8] Baayen RH, Milin P, Filipović Durdević D, Hendrix P, Marelli M (2011). An amorphous model for morphological processing in visual comprehension based on naive discriminative learning. Psychological Review.

[CR9] Baayen RH, Shaoul C, Willits J, Ramscar M (2016). Comprehension without segmentation: A proof of concept with naive discriminative learning. Language, Cognition, and Neuroscience.

[CR10] Beard R (1977). On the extent and nature of irregularity in the lexicon. Lingua.

[CR11] Beyersmann E, Casalis S, Ziegler JC, Grainger J (2015). Language proficiency and morpho-orthographic segmentation. Psychonomic bulletin & Review.

[CR12] Beyersmann E, Ziegler JC, Castles A, Coltheart M, Kezilas Y, Grainger J (2016). Morpho-orthographic segmentation without semantics. Psychonomic Bulletin & Review.

[CR13] Beygelzimer, A., Kakadet, S., Langford, J., Arya, S., Mount, D., & Li, S. (2018). FNN: Fast Nearest Neighbor Search Algorithms and Applications. R package version 1.1.2.1.

[CR14] Bitan T, Kaftory A, Meiri-Leib A, Eviatar Z, Peleg O (2017). Phonological ambiguity modulates resolution of semantic ambiguity during reading: An fMRI study of Hebrew. Neuropsychology.

[CR15] Blevins JP (2003). Stems and paradigms. Language.

[CR16] Blevins JP (2006). Word-based morphology. Journal of Linguistics.

[CR17] Blevins JP (2016). Word and paradigm morphology.

[CR18] Bojanowski P, Grave E, Joulin A, Mikolov T (2017). Enriching word vectors with subword information. Transactions of the Association for Computational Linguistics.

[CR19] Botha, J. , & Blunsom, P. (2014). Compositional morphology for word representations and language modelling. In *International Conference on Machine Learning* (pp. 1899–1907).

[CR20] Butz MV, Kutter EF (2016). How the mind comes into being: Introducing cognitive science from a functional and computational perspective.

[CR21] Cassani, G., Chuang, Y.-Y., & Baayen, R. H. (2019). On the semantics of nonwords and their lexical category. Journal of Experimental Psychology: Learning, Memory, and Cognition, 1–18. 10.1037/xlm0000747 31318232

[CR22] Chen, X., Xu, L., Liu, Z., Sun, M., & Luan, H.-B. (2015). Joint learning of character and word embeddings. In *IJCAI* (pp. 1236–1242).

[CR23] Chuang, Y.-Y., Lõo, K., Blevins, J. P., & Baayen, R. H. (2019). Estonian case inflection made simple. A case study in word and paradigm morphology with linear discriminative learning. *PsyArXiv*, 1–19.

[CR24] Cieliebak, M., Deriu, J. M., Egger, D., & Uzdilli, F. (2017). A Twitter corpus and benchmark resources for German sentiment analysis. In *Proceedings of the Fifth International Workshop on Natural Language Processing for Social Media, Valencia, Spain* (pp. 45–51).

[CR25] Cohen C (2014). Probabilistic reduction and probabilistic enhancement. Morphology.

[CR26] Coltheart M, Curtis B, Atkins P, Haller M (1993). Models of reading aloud: Dual-route and parallel-distributed-processing approaches. Psychological Review.

[CR27] Coltheart M, Rastle K, Perry C, Langdon R, Ziegler J (2001). The DRC model: A model of visual word recognition and reading aloud. Psychological Review.

[CR28] Cotterell, R., & Schütze, H. (2015). Morphological word-embeddings. In *Proceedings of the 2015 Conference of the North American chapter of the association for computational linguistics: Human language technologies* (pp. 1287–1292).

[CR29] Csardi G, Nepusz T (2006). The igraph software package for complex network research. Interjournal.

[CR30] Dell G (1986). A spreading-activation theory of retrieval in sentence production. Psychological Review.

[CR31] Dell GS, Gordon JK (2003). Neighbors in the lexicon: Friends or foes?. Phonetics and phonology in language comprehension and production: Differences and similarities.

[CR32] Deriu, J., Lucchi, A., De Luca, V., Severyn, A., Müller, S., Cieliebak, M., ..., Jaggi, M. (2017). Leveraging large amounts of weakly supervised data for multi-language sentiment classification. In *Proceedings of the 26th international conference on World Wide Web (WWW-2017), Perth, Australia* (pp. 1045–1052).

[CR33] Embick D, Poeppel D (2015). Towards a computational (IST) neurobiology of language: Correlational, integrated and explanatory neurolinguistics. Language, Cognition and Neuroscience.

[CR34] Feldman LB, O’Connor PA, Moscoso del Prado MF (2009). Early morphological processing is morpho-semantic and not simply morpho-orthographic: Evidence from the masked priming paradigm. Psychonomic Bulletin & Review.

[CR35] Fitneva SA, Christiansen MH, Monaghan P (2009). From sound to syntax: Phonological constraints on children’s lexical categorization of new words. Journal of Child Language.

[CR36] Forster, K. I. (1976). Accessing the mental lexicon. In R. J. Wales, & E. Walker (Eds.) *New approaches to language mechanisms. A collection of psycholinguistic studies* (pp. 257–287). Amsterdam: North-Holland.

[CR37] Frege G (1879). Begriffsschrift, a formula language, modeled upon that of arithmetic, for pure thought. From Frege to Gö,del: A source book in mathematical logic.

[CR38] Gahl S, Yao Y, Johnson K (2012). Why reduce? phonological neighborhood density and phonetic reduction in spontaneous speech. Journal of Memory and Language.

[CR39] Gibson, J.J. (1977). The theory of affordances. Perceiving, acting, and knowing.

[CR40] Gonnerman LM, Seidenberg MS, Andersen ES (2007). Graded semantic and phonological similarity effects in priming: Evidence for a distributed connectionist approach to morphology. Journal of Experimental Psychology: General.

[CR41] Grainger J, Jacobs AM (1996). Orthographic processing in visual word recognition: A multiple read-out model. Psychological Review.

[CR42] Gries, S. T. (2004). Shouldn’t it be breakfunch? a quantitative analysis of blend structure in English. *Linguistics*, 639–668.

[CR43] Halle, M., & Marantz, A. (1993). Distributed morphology and the pieces of inflection. In K. Hale, & S. J. Keyser (Eds.) *The view from building 20: Essays in Linguistics in Honor of Sylvain Bromberger, volume 24 of Current Studies in Linguistics* (pp. 111–176). Cambridge: MIT Press.

[CR44] Hannun, A., Case, C., Casper, J., Catanzaro, B., Diamos, G., Elsen, E., ..., et al. (2014). Deep speech: Scaling up end-to-end speech recognition. arXiv:1412.5567.

[CR45] Harm MW, Seidenberg MS (2004). Computing the meanings of words in reading: Cooperative division of labor between visual and phonological processes. Psychological Review.

[CR46] Hendrix, P., & Sun, C.C. (2020). A word or two about nonwords: Frequency, semantic neighborhood density, and orthography-to-semantics consistency effects for nonwords in the lexical decision task. Journal of Experimental Psychology. Learning, Memory, and Cognition, 1–28. 10.1037/xlm0000819 31999159

[CR47] Hickok G (2014). The architecture of speech production and the role of the phoneme in speech processing. Language, Cognition and Neuroscience.

[CR48] Hockett C (1954). Two models of grammatical description. Word.

[CR49] Hornstein N (1995). Logical form: From GB to minimalism.

[CR50] Imai M, Kita S (2014). The sound symbolism bootstrapping hypothesis for language acquisition and language evolution. Philosophical transactions of the Royal Society London B - Biological Sciences.

[CR51] Ivens, S. H., & Koslin, B. L. (1991). Demands for reading literacy require new accountability methods. Touchstone Applied Science Associates.

[CR52] Jared D, Ashby J, Agauas SJ, Levy BA (2016). Phonological activation of word meanings in grade 5 readers. Journal of Experimental Psychology: Learning, Memory, and Cognition.

[CR53] Jared D, Bainbridge S (2017). Reading homophone puns: Evidence from eye tracking. Canadian Journal of Experimental Psychology/Revue canadienne de psychologie expérimentale.

[CR54] Jared D, O’Donnell K (2017). Skilled adult readers activate the meanings of high-frequency words using phonology: Evidence from eye tracking. Memory & Cognition.

[CR55] Jones MN, Mewhort DJK (2007). Representing word meaning and order information in a composite holographic lexicon. Psychological Review.

[CR56] Kappel D, Habenschuss S, Legenstein R, Maass W (2015). Network plasticity as Bayesian inference. PLoS Computational Biology.

[CR57] Kappel, D., Legenstein, R., Habenschuss, S., Hsieh, M., & Maass, W. (2017). Reward-based stochastic self-configuration of neural circuits. arXiv:1704.04238.

[CR58] Kemps R, Ernestus M, Schreuder R, Baayen RH (2004). Processing reduced word forms: The suffix restoration effect. Brain and Language.

[CR59] Kemps R, Ernestus M, Schreuder R, Baayen RH (2005). Prosodic cues for morphological complexity: The case of Dutch noun plurals. Memory and Cognition.

[CR60] Kemps R, Wurm LH, Ernestus M, Schreuder R, Baayen RH (2005). Prosodic cues for morphological complexity in Dutch and English. Language and Cognitive Processes.

[CR61] Keuleers E, Brysbaert M (2010). Wuggy: A multilingual pseudoword generator. Behavior Research Methods.

[CR62] Keuleers E, Lacey P, Rastle K, Brysbaert M (2012). The British Lexicon Project: Lexical decision data for 28,730 monosyllabic and disyllabic English words. Behavior Research Methods.

[CR63] Köhler W (1929). Gestalt psychology.

[CR64] Kuperman V, Pluymaekers M, Ernestus M, Baayen RH (2006). Morphological predictability and acoustic salience of interfixes in Dutch compounds. JASA.

[CR65] Kuperman V, Schreuder R, Bertram R, Baayen RH (2009). Reading of multimorphemic Dutch compounds: Towards a multiple route model of lexical processing. Journal of Experimental Psychology: HPP.

[CR66] Landauer T, Dumais S (1997). A solution to Plato’s problem: The latent semantic analysis theory of acquisition, induction and representation of knowledge. Psychological Review.

[CR67] Levelt W, Roelofs A, Meyer AS (1999). A theory of lexical access in speech production. Behavioral and Brain Sciences.

[CR68] Linke M, Broeker F, Ramscar M, Baayen RH (2017). Are baboons learning “orthographic” representations? Probably not. PLOS-ONE.

[CR69] Long, R. (2018). Enhancing the TASA corpus for analysis using naive discriminative learning. Unpublished MA thesis, University of Tuebingen.

[CR70] Luong, T., Socher, R., & Manning, C. (2013). Better word representations with recursive neural networks for morphology. In *Proceedings of the seventeenth conference on computational natural language learning* (pp. 104–113).

[CR71] Marantz A (2013). No escape from morphemes in morphological processing. Language and Cognitive Processes.

[CR72] Marelli, M., Amenta, S., & Crepaldi, D. (2014). Semantic transparency in free stems: The effect of orthography-semantics consistency in word recognition. Quarterly Journal of Experimental Psychology, in press. 10.1080/17470218.2014.959709 25269473

[CR73] Matthews PH (1974). Morphology an introduction to the theory of word structure.

[CR74] Matthews PH (1991). Morphology an introduction to the theory of word structure.

[CR75] Maurer D, Pathman T, Mondloch CJ (2006). The shape of boubas: Sound-shape correspondences in toddlers and adults. Developmental Science.

[CR76] McCarthy JJ (1981). A prosodic theory of non-concatenative morphology. Linguistic Inquiry.

[CR77] McClelland JL, Elman JL (1986). The TRACE model of speech perception. Cognitive Psychology.

[CR78] McClelland JL, Rumelhart DE (1981). An interactive activation model of context effects in letter perception: Part I. An account of the basic findings. Psychological Review.

[CR79] Melamud, O., McClosky, D., Patwardhan, S., & Bansal, M. (2016). The role of context types and dimensionality in learning word embeddings, pp. 1–11. arXiv:1601.00893v2.

[CR80] Mikolov, T., Sutskever, I., Chen, K., Corrado, G. S., & Dean, J. (2013). Distributed representations of words and phrases and their compositionality. In *Advances in neural information processing systems* (pp. 3111–3119).

[CR81] Milin P, Feldman LB, Ramscar M, Hendrix P, Baayen RH (2017). Discrimination in lexical decision. PLOS-one.

[CR82] Mirković J, MacDonald MC, Seidenberg MS (2005). Where does gender come from? Evidence from a complex inflectional system. Language and Cognitive Processes.

[CR83] Montague, R. (1973). The proper treatment of quantification in ordinary English. In *Approaches to natural language* (pp. 221–242): Springer.

[CR84] Newman RL, Jared D, Haigh CA (2012). Does phonology play a role when skilled readers read high-frequency words? Evidence from ERPS. Language and Cognitive Processes.

[CR85] Norris D (2006). The Bayesian reader: Explaining word recognition as an optimal Bayesian decision process. Psychological Review.

[CR86] Norris D, McQueen J (2008). Shortlist B: A Bayesian model of continuous speech recognition. Psychological Review.

[CR87] Norris DG (1994). Shortlist: A connectionist model of continuous speech recognition. Cognition.

[CR88] Oord, A. v. d., Dieleman, S., Zen, H., Simonyan, K., Vinyals, O., Graves, A., ..., Kavukcuoglu, K. (2016). Wavenet: A generative model for raw audio. arXiv:1609.03499.

[CR89] Perrone-Bertolotti M, Kujala J, Vidal JR, Hamame CM, Ossandon T, Bertrand O, Minotti L, Kahane P, Jerbi K, Lachaux J-P (2012). How silent is silent reading? Intracerebral evidence for top-down activation of temporal voice areas during reading. Journal of Neuroscience.

[CR90] Pham H, Baayen RH (2015). Vietnamese compounds show an anti-frequency effect in visual lexical decision. Language, Cognition, and Neuroscience.

[CR91] Plaut DC, Gonnerman LM (2000). Are non-semantic morphological effects incompatible with a distributed connectionist approach to lexical processing?. Language and Cognitive Processes.

[CR92] Pluymaekers M, Ernestus M, Baayen RH (2005). Lexical frequency and acoustic reduction in spoken Dutch. Journal of the Acoustical Society of America.

[CR93] Qiu, S., Cui, Q., Bian, J., Gao, B., & Liu, T.-Y. (2014). Co-learning of word representations and morpheme representations. In *Proceedings of COLING 2014, the 25th International Conference on Computational Linguistics: Technical Papers* (pp. 141–150).

[CR94] Rastle K, Davis M (2008). Morphological decomposition based on the analysis of orthography. Language and Cognitive Processes.

[CR95] Russell B (1905). On denoting. Mind.

[CR96] Russell B (1942). An inquiry into meaning and truth.

[CR97] Sapir E (1929). A study in phonetic symbolism. Journal of Experimental Psychology.

[CR98] Schmid, H. (1995). Improvements in part-of-speech tagging with an application to German. In *Proceedings of the ACL SIGDAT-workshop*, Dublin, Ireland.

[CR99] Schmidtke, D., Matsuki, K., & Kuperman, V. (2017). Surviving blind decomposition: A distributional analysis of the time course of complex word recognition. *Journal of Experimental Psychology: Learning, Memory and Cognition*. 10.1037/xlm0000411 PMC5659973 28447810

[CR100] Seidenberg MS, Gonnerman LM (2000). Explaining derivational morphology as the convergence of codes. Trends in Cognitive Sciences.

[CR101] Seidenberg MS, McClelland JL (1989). A distributed, developmental model of word recognition and naming. Psychological Review.

[CR102] Sering, K., Stehwien, N., & Gao, Y. (2019). create_vtl_corpus: Synthesizing a speech corpus with vocaltractlab (version v1.0.0). Zenodo. 10.5281/zenodo.2548895.

[CR103] Sering, T., Milin, P., & Baayen, R. H. (2018). Language comprehension as a multiple label classification problem. *Statistica Neerlandica*, 1–15.

[CR104] Shaoul C, Westbury C (2010). Exploring lexical co-occurrence space using hiDEx. Behavior Research Methods.

[CR105] Silver D, Huang A, Maddison CJ, Guez A, Sifre L, Van Den Driessche G, Schrittwieser J, Antonoglou I, Panneershelvam V, Lanctot M (2016). Mastering the game of go with deep neural networks and tree search. Nature.

[CR106] Skousen, R. (2000). Analogical modeling and quantum computing. Los Alamos National Laboratory <http://arXiv.org>.

[CR107] Smolka E, Preller KH, Eulitz C (2014). ‘verstehen’(‘understand’) primes ‘stehen’(‘stand’): Morphological structure overrides semantic compositionality in the lexical representation of German complex verbs. Journal of Memory and Language.

[CR108] Smolka E, Zwitserlood P, Rösler F (2007). Stem access in regular and irregular inflection: Evidence from German participles. Journal of Memory and Language.

[CR109] Stump G (2001). Inflectional morphology: A theory of paradigm structure.

[CR110] Taft M, Forster KI (1975). Lexical storage and retrieval of prefixed words. Journal of Verbal Learning and Verbal Behavior.

[CR111] Taft M, Forster KI (1976). Lexical storage and retrieval of polymorphemic and polysyllabic words. Journal of Verbal Learning and Verbal Behavior.

[CR112] Ten Bosch, L., Boves, L., & Ernestus, M. (2015). Diana, an end-to-end computational model of human word comprehension. In *Proceedings of the 18th International Congress of Phonetic Sciences, Glasgow. Scottish Consortium for ICPhS*.

[CR113] Tomaschek F, Hendrix P, Baayen RH (2018). Strategies for addressing collinearity in multivariate linguistic data. Journal of Phonetics.

[CR114] Tucker, B. V., Brenner, D., Danielson, D. K., Kelley, M. C., Nenadić, F., & Sims, M. (2018). The massive auditory lexical decision (MALD) database. *Behavior research methods*, 1–18. 10.3758/s13428-018-1056-1 29916041

[CR115] Ussishkin A (2005). A fixed prosodic theory of nonconcatenative templatic morphology. Natural Language & Linguistic Theory.

[CR116] Velan H, Frost R, Deutsch A, Plaut DC (2005). The processing of root morphemes in Hebrew: Contrasting localist and distributed accounts. Language and Cognitive Processes.

[CR117] Veríssimo, J. (2018). Taking it a level higher: The LEIA model of complex word recognition. Poster presented at AMLaP 2018, Berlin.

[CR118] Vitevitch MS, Luce PA (1998). When words compete: Levels of processing in perception of spoken words. Psychological Science.

[CR119] Vitevitch MS, Luce PA (1999). Probabilistic phonotactics and neighborhood activation in spoken word recognition. Journal of Memory and Language.

[CR120] Vitevitch MS, Luce PA (2004). A web-based interface to calculate phonotactic probability for words and nonwords in English. Behavior Research Methods, Instruments, & Computers.

[CR121] Vitevitch MS, Luce PA, Charles-Luce J, Kemmerer D (1997). Phonotactics and syllable stress: Implications for the processing of spoken nonsense words. Language and Speech.

[CR122] Westbury C (2005). Implicit sound symbolism in lexical access: Evidence from an interference task. Brain and Language.

[CR123] Westbury C, Hollis G, Sidhu DM, Pexman PM (2018). Weighing up the evidence for sound symbolism: Distributional properties predict cue strength. Journal of Memory and Language.

[CR124] Widrow, B., & Hoff, M. E. (1960). Adaptive switching circuits. *1960 WESCON Convention Record Part IV*, 96–104.

[CR125] Wong KFE, Chen H-C (1999). Orthographic and phonological processing in reading Chinese text: Evidence from eye fixations. Language and Cognitive Processes.

[CR126] Zwitserlood, P. (2018). Processing and representation of morphological complexity in native language comprehension and production. In G. E. Booij (Ed.) *The construction of words. Advances in construction morphology* (pp. 583–602): Springer.

