# Supplementary material for: The processing of pseudoword form and meaning in production and comprehension: A computational modeling approach using linear discriminative learning
Source: Behav Res Methods. 2020 May 6;53(3):945–76. doi: 10.3758/s13428-020-01356-w (PMC8219637; doi:10.3758/s13428-020-01356-w)
Supplement: Supplementary file 1 — (PDF 283 KB) [file 13428_2020_1356_MOESM1_ESM.pdf]

Supplementary materials

1 Predictors for words

1.1 Distributions of predictors for words

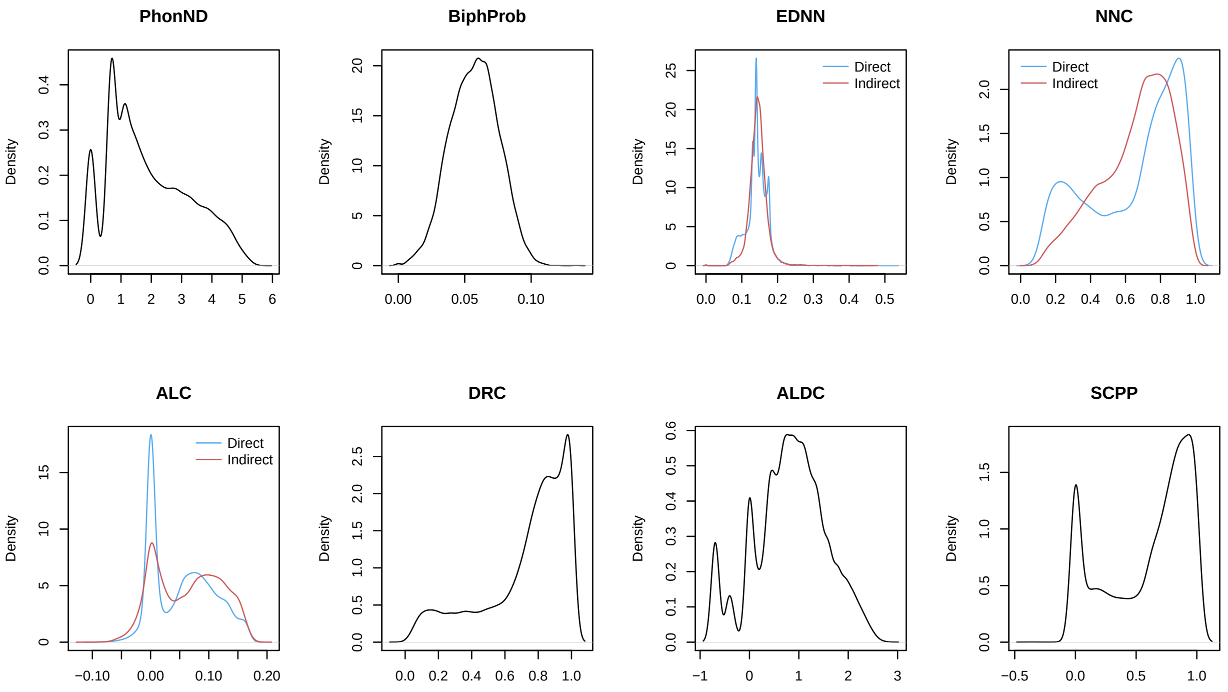

Figure 1: The density plots of all the predictors for words. `PhonND` and `prodallLev` log-transformed; `BiphProb` and the `EDNN` measures for the two routes were transformed by taking square roots.

1.2 Comparison between predictors for pseudowords and words

Table 1: Mean values of the predictors for pseudowords and words.

	PhonND	BiphProb	EDNN$_1$	EDNN$_2$	NNC$_1$	NNC$_2$	ALC$_1$	ALC$_2$	DRC	ALDC	SCPP
pseudoword	5.83	0.003	1.69	1.27	0.29	0.29	0.01	0.01	0.74	4.83	0.49
word	16.07	0.004[a]	0.02	0.02	0.65	0.66	0.05	0.06	0.73	2.88	0.60

a: `BiphProb` is not available for five single-phone words.

2 More model summaries

2.1 Predict pseudoword durations with both phonological and LDL predictors

Table 2: GAM fitted to square-root transformed pseudoword duration with both the phonological and the LDL measures as predictors. s: thin plate regression spline smooth.

A. parametric coefficients	Estimate	Std. Error	t-value	p-value
Intercept	24.0275	0.0231	1041.7	< 0.0001
IsInfl:TRUE	0.3401	0.0365	9.34	< 0.0001
B. smooth terms	edf	Ref.df	F-value	p-value
s(PC1)	3.428	4.330	117.1	< 0.0001
s(PC2)	1.003	1.005	161.5	< 0.0001
s(PC3)	1.000	1.000	688.0	< 0.0001
s(ALDC)	4.607	5.697	543.3	< 0.0001
s(SCPP)	5.853	7.040	103.3	< 0.0001
s(PhonND)	1.001	1.003	641.5	< 0.0001
s(BiphProb)	2.985	3.806	3.6	< 0.01

-ML = 18666, R^2(adj) = 0.621

2.2 Predict pseudoword reaction times with both phonological and LDL predictors

Table 3: GAMM fitted to Box-Cox transformed pseudoword RT with both the phonological and the LDL measures as predictors. s: thin plate regression spline smooth.

A. parametric coefficients	Estimate	Std. Error	t-value	p-value
Intercept	4973.06	30.36	163.8	< 0.0001
IsInfl:TRUE	-15.86	6.92	-2.3	< 0.05
B. smooth terms	edf	Ref.df	F-value	p-value
s(PC1)	3.192	3.522	27.288	< 0.0001
s(PC2)	1.005	1.007	40.812	< 0.0001
s(PC3)	1.023	1.031	98.960	< 0.0001
s(ALDC)	4.879	5.314	139.92	< 0.0001
s(SCPP)	5.213	5.652	29.30	< 0.0001
s(PhonND)	3.783	4.096	98.63	< 0.0001
s(BiphProb)	3.204	3.538	4.27	< 0.005
s(Subject)	228.00	230.00	166.75	< 0.0001
s(Pseudoword)	5688.25	9564	5.113	< 0.0001

fREML = 158490, R^2(adj) = 0.325

3 Appendix A

3.1 Density plots of the LDL predictors derived from the model trained with Word2Vec embeddings

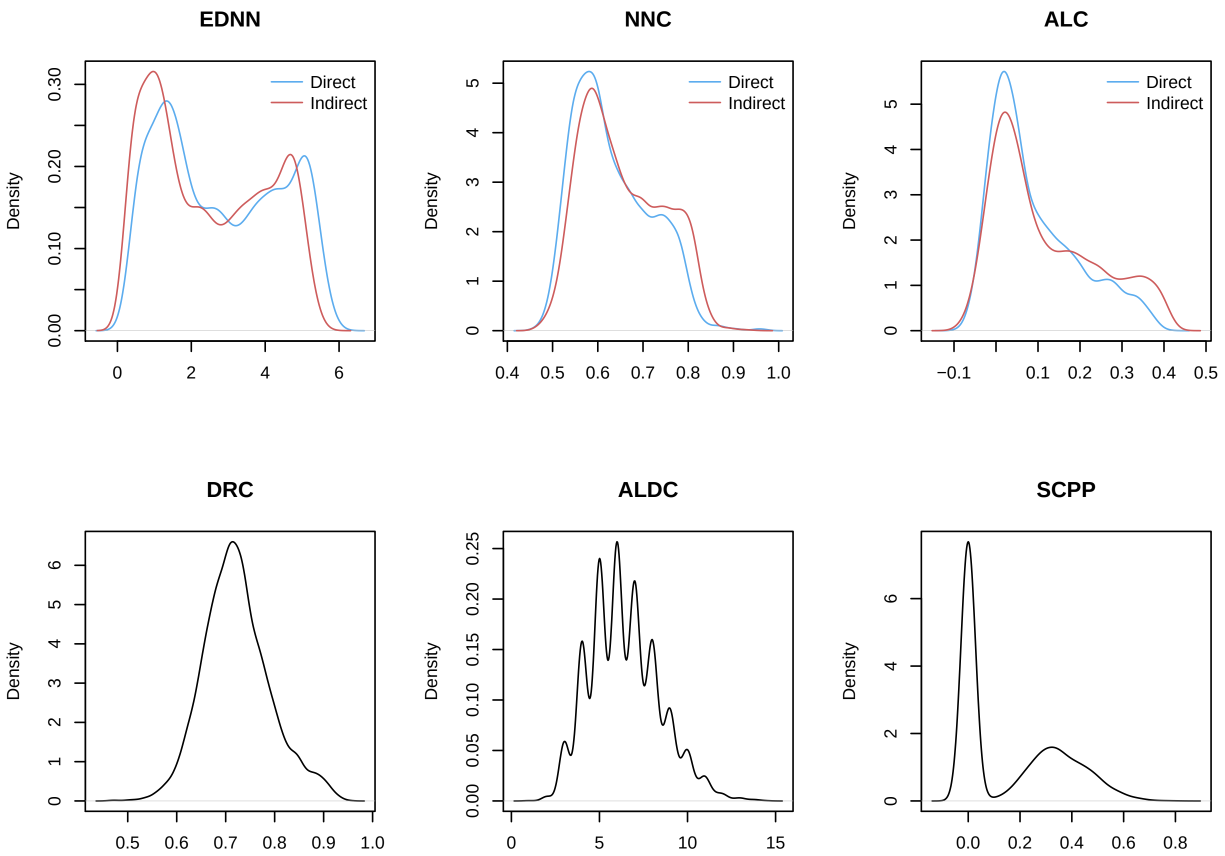

Figure 2: The density plots of the LDL predictors derived from the model trained with `Word2Vec` embeddings. The `EDNN` measures for the two routes were log-transformed, and the two `NNC` measures were transformed by taking square roots.

3.2 PCA loadings for the Word2Vec-based semantic measures

Table 4: PCA loadings for the Word2Vec-based semantic measures.

	PC1	PC2	PC3
EDNN	0.50	-0.43	0.72
NNC	-0.53	0.19	0.64
ALC	-0.54	0.10	0.20
DRC	-0.42	-0.88	0.20

3.3 The partial effects of the Word2Vec-based LDL measures on pseudoword duration

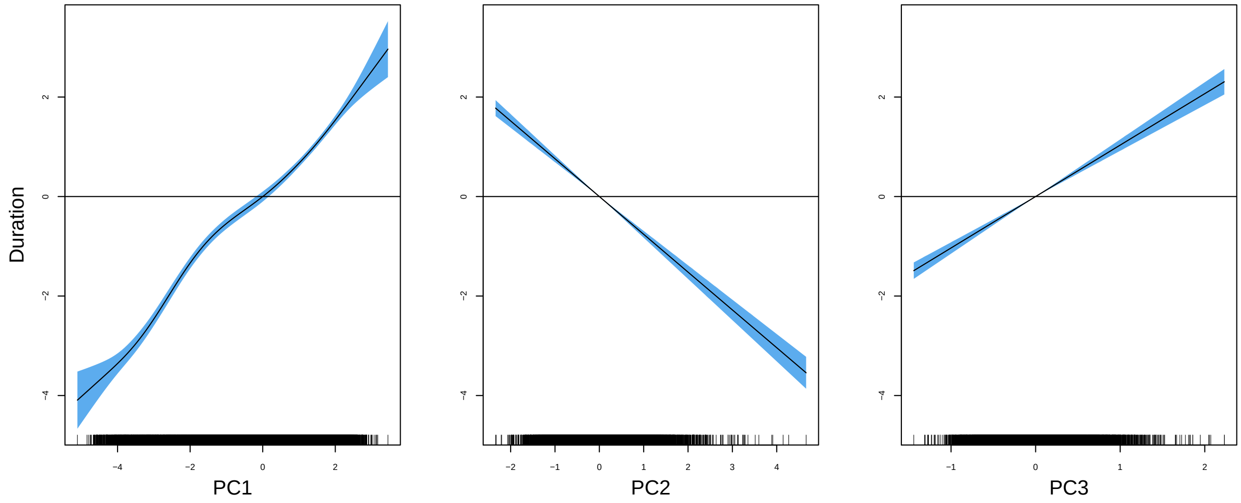

Figure 3: The partial effects of the the Word2Vec-based LDL measures on square-root transformed pseudoword duration.
